# Fungal Screening for Potential PET Depolymerization

**DOI:** 10.3390/polym15061581

**Published:** 2023-03-22

**Authors:** Lusiane Malafatti-Picca, Elaine Cristina Bucioli, Michel Ricardo de Barros Chaves, Aline Machado de Castro, Érika Valoni, Valéria Maia de Oliveira, Anita Jocelyne Marsaioli, José Silvio Govone, Dejanira de Franceschi de Angelis, Michel Brienzo, Derlene Attili-Angelis

**Affiliations:** 1 Environmental Studies Center (CEA), São Paulo State University (UNESP), Av. 24-A, 1515, Bela Vista, Rio Claro 13506-900, SP, Brazil; lusianepicca@gmail.com (L.M.-P.);; 2 Coordination of Natural Sciences, Federal University of Maranhão (UFMA), Av. João Alberto, 700, Bacabal 65700-000, MA, Brazil; 3 Department of Biotechnology, R&D Center, PETROBRAS, Av. Horácio Macedo, 950, Ilha do Fundão, Rio de Janeiro 21941-915, RJ, Brazil; 4 Division of Microbial Resources, CPQBA, State University of Campinas (Unicamp), Rua Alexandre Cazellato, 999, Paulínia 13148-218, SP, Brazil; 5 Institute of Chemistry, State University of Campinas (Unicamp), P.O. Box 6154, Campinas 13084-971, SP, Brazil; 6 Department of Biochemistry and Microbiology, São Paulo State University (UNESP), Av. 24-A, 1515, Bela Vista, Rio Claro 13506-900, SP, Brazil; 7 Institute For Research in Bioenergy (IPBEN), São Paulo State University (UNESP), R. 10, 2527, Santana, Rio Claro 13500-230, SP, Brazil

**Keywords:** biodegradation, enzymatic catalysis, polymers, terephthalic acid

## Abstract

Approximately 400 billion PET bottles are produced annually in the world, of which from 8 to 9 million tons are discarded in oceans. This requires developing strategies to urgently recycle them. PET recycling can be carried out using the microbial hydrolysis of polymers when monomers and oligomers are released. Exploring the metabolic activity of fungi is an environmentally friendly way to treat harmful polymeric waste and obtain the production of monomers. The present study addressed: (i) the investigation of potential of strains with the potential for the depolymerization of PET bottles from different manufacturers (crystallinity of 35.5 and 10.4%); (ii) the search for a culture medium that favors the depolymerization process; and (iii) gaining more knowledge on fungal enzymes that can be applied to PET recycling. Four strains (from 100 fungal strains) were found as promising for conversion into terephthalic acid from PET nanoparticles (npPET): *Curvularia trifolii* CBMAI 2111, *Trichoderma* sp. CBMAI 2071, *Trichoderma atroviride* CBMAI 2073, and *Cladosporium cladosporioides* CBMAI 2075. The fermentation assays in the presence of PET led to the release of terephthalic acid in concentrations above 12 ppm. Biodegradation was also confirmed using mass variation analyses (reducing mass), scanning electron microscopy (SEM) that showed evidence of material roughness, FTIR analysis that showed band modification, enzymatic activities detected for lipase, and esterase and cutinase, confirmed by monomers/oligomers quantification using high performance liquid chromatography (HPLC-UV). Based on the microbial strains PET depolymerization, the results are promising for the exploration of the selected microbial strain.

## 1. Introduction

Despite the environmental damage caused by plastic, its current application in human society has become so diverse that a short-term replacement is unlikely. In 2018, the world production of polymers reached 360 million tons (Mtons). If the current treatment (i.e., production and disposal) of plastics persists by 2050, it is believed that 12 Mtons of this material will be deposited in landfills or the natural environment [1]. Europe alone was responsible for causing 62 Mtons of this global amount to be available, of which 7.7% comprised polyethylene terephthalate (PET), a thermoplastic with an excellent mechanical and thermal resistance [2,3]. The initiatives by the European Parliament intended to increase PET recycling with the support of international companies and the promise to step up recycling. However, a decline in the uptake by the selective collection of the polymer has been observed, threatening the goal of the estimated amount for 2025 [4]. It is assumed that, only in 2021, will the number of produced bottles reach 583.3 billion units worldwide [5], which is a serious matter of concern for the environment.

The great persistence and low (bio-) degradability reported under natural conditions are responsible for the huge environmental impact of inadequate PET disposal. The material may require thousands of years to degrade [6,7]. The shortage of production associated with excessive consumption and the timid reuse of PET bottles requires research and development aimed at remediating this recalcitrant material. It is estimated that the amount of plastic packaging on the market may quickly become equal to the quantity discarded, reinforcing the urgency for establishing new recycling strategies for this material [8].

The bottles for beverage and food recycling relates to the production of fibers and special films, laminated and thermoformed, as well as textiles [3]. PET recycling routes may be mechanical, chemical, or energetic [9]. Among them, chemical recycling emerges as a more interesting alternative because it prevents the deposition of solid waste in the environment, besides providing monomers to obtain new bottles and packaging [10,11]. The types of chemical recycling are glycolysis, methanolysis, aminolysis, ammonolysis, and hydrolysis. The first has been the most used by the industry despite the high cost associated with separating and refining the products in the mixture [12]. The studies on the depolymerization of PET search for innovative aspects and are certainly of great scientific, economic, and industrial importance [13]. Alternatively, microorganism-mediated polymer hydrolysis occurs with the advantage of not requiring solvents and reagents that usually increase purification costs [14].

Microbial hydrolytic enzymes such as lipases (EC 3.1.1.3), esterases (EC 3.1.1.6 and EC 3.1.1.2), and cutinases (EC 3.1.1.74) can assist in the biodegradation of the hydrophobic polymer [15,16,17]. Several studies have addressed the use of microorganisms or their enzymes in PET depolymerization processes [18,19,20]. Recently, the species *Ideonella sakaiensis* (Burkholderiales, Proteobacteria), isolated in Japan, has been in the limelight due to the selectivity and efficiency of its enzymes in 18 h of activity [21]. Despite the satisfactory activity of some hydrolases, the microbial conversion of PET is far from complete. It is still not clear in which species or phyla these enzymes can be mainly found. According to Lear et al. [22], these questions represent important notes that must be answered, which justifies further studies.

Fungi are excellent degraders of organic matter, especially natural polymers, as they grow under adverse conditions of pH and humidity. In addition, mycelial development favors the colonization of the substrate, which, together with its multienzyme system, speeds up the degradation of several compounds [23]. The literature highlights cutinases from *Fusarium solani* [24] and esterases from *Cladosporium cladosporioides* [25] and *Penicillium citrinum* [26], as well as lipases from *Aspergillus oryzae* [27], which have the potential to increase the hydrophilicity of the PET polymer. Recently, Malafatti-Picca et al. [18] selected nine strains of remarkable hydrolytic activity for converting PET. However, most of the reports still focus only on bacterial species, while fungi remain unexplored for such an application. This fact is evinced in the literature as highlighted in the reviews performed by Taniguchi et al. [3] and Ru et al. [28].

Several methods are used to evaluate microbial activity on polymers. High-Performance Liquid Chromatography (HPLC) is used to study microbial activity on polymers, allowing for the quantification of the released terephthalic acid (PTA) monomers and bis(2-hydroxyethyl) terephthalate (BHET) and mono(hydroxyethyl) terephthalate (MHET) oligomers. Although efficient, the methodology is time-consuming and requires specific infrastructure and adequate room [24,28]. Miniaturized screening approaches to detect polyester hydrolases are considered of great value because they are quick and helpful for the development of sustainable methodologies. Wei et al. [29], for example, implemented a high-performance screening technique with fluorescence detection (HTS-fluorescence) for the quantification of terephthalic acid from PET nanoparticles (npPET) by converting PTA to the free radical 2-hydroxyterephthalate (HOTP). The assays of this investigation were carried out with recombinant cutinases of *Thermobifida fusca* (Actinomycetales, Bacteria). Addressing the same principle, Chaves et al. [30] reported the bacteria, filamentous fungi, and yeasts with the potential for depolymerization of npPET in PTA, although using the entire cell structure of the tested isolates, not just the isolated enzymes.

Focusing on fungal strains from hydrocarbon-associated environments, this work evaluated their hydrolase production potential for npPET assimilation using microscale assays. The effect of the strains with the best conversion rates on fragments of PET bottles from two brands and different production technologies, “plant-bottle” and conventional, was evaluated. For this purpose, the analyses of PTA, BHET, and MHET quantification through HPLC-UV after liquid–liquid extraction, as well as total proteins and hydrolase activity (lipase and esterase), were carried out on fermented liquid (free of mycelium). On PET fragments, the analyses included weight variation, scanning electron microscopy (SEM), and infrared spectroscopy (FTIR). The proposal of a culture medium to provide greater PET conversion into PTA and oligomers was another contribution of the present study.

## 2. Material and Methods

### 2.1. Microorganisms

One hundred fungal strains were investigated in this work, which was the same number Malafatti-Picca [18] used in their study. The strains were obtained from a culture collection of interest to the petrochemical industry deposited in the Brazilian Collection of Environmental and Industrial Microorganisms (CBMAI, Unicamp, SP, Brazil) and/or preserved in the working research collection of the laboratory.

#### 2.1.1. Enzymatic Assays with PET nanoparticles

The isolates were cultivated in Potato Dextrose Agar (PDA) purchased from Kasvi, Spain, at 28 °C for seven days. The analyses were performed in 96 well plates with a black background divided into enzymatic assays and negative, positive, and microbial control, as indicated in Table 1.

Afterward, the plates were incubated at 28 °C and agitated at 200 rpm. The enzyme activities were monitored at zero, five, ten, and fifteen days. The following reagents obtained from Sigma-Aldrich, (Saint Louis, MO, USA) were added per well, in the following order: 30 µL of a 2% H_2_O_2_ solution (CAS 7722-84-1), 20 µL of 2 mM EDTA (CAS 13235-36-4), and 20 µL of 3 mM Fe_2_SO_4_·7H_2_O (CAS 7782-63-0), resulting, respectively, in the final concentrations in the wells of 0.0026 mM, 0.2 mM, and 0.3 mM. After that, the plates were incubated at room temperature for 25 min for the conversion of terephthalic acid into HOTP, then the fluorescence was revealed [30].

The fluorescence was observed at wavelengths λex = 328 nm (excitation) and λem = 421 nm (emission) using a TM 2300 EnSpire^®^ multimode plate reader (PerkinElmer, Waltham, MA, USA). The conversion calculations from PTA into HOTP were performed, as mentioned by Chaves et al. [30] and Malafatti-Picca et al. [18].

**Figure 1:**
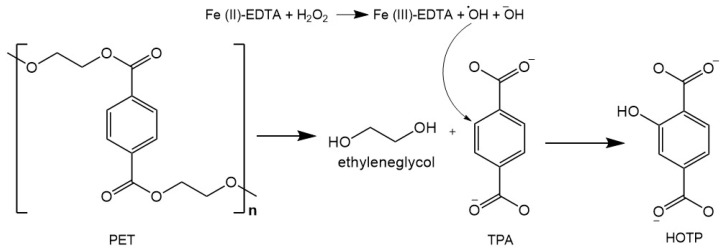
Conversion reaction of terephthalic acid (PTA) into 2-hidroxyterephthalate (HOTP). Note. Reagents added: 30 µL 2% H_2_O_2_; 20 µL 2mM EDTA; and 20 µL Fe_2_SO_4_·7H_2_O 3 mM. Adapted from Chaves et al. [30].

Figure 1 represents the PTA modification scheme into HOTP after adding the fluorescence developers.

#### 2.1.2. Assays to Detect Cutinase Production

##### Plate Tests with Cutin and Polycaprolactone (PCL) as the Sole Carbon Sources

Agar blocks (3.0 mm of diameter) containing fungal mycelium grown for seven days in PDA at 28 °C were transferred to the center of two different Petri dishes (90 mm × 15 mm) with two configurations: (i) minimal mineral medium supplemented with cutin (extracted from Fuji apple peel, according to the methodology proposed by Macedo and Pio [32]); and (ii) minimal mineral medium supplemented with polycaprolactone (Sigma Aldrich^®^, Saint Louis, MO, USA—average molar mass 10,000 g·mol^−1^). The formulations of the culture media were the following:

1. *Cutin:* 3.0 g NaNO_3_ (CAS 7631-99-4); 1.0 g K_2_HPO_4_ (CAS 7758-11-4); 0.5 g KCl (CAS 7447-40-7); 0.01 g FeSO_4_·7H_2_O (CAS 7782-63-0); 0.2% cutin; 17 g agar (CAS 9002-18-0); and 1000 mL distilled water [33,34].
2. Polycaprolactone: 4 g (NH_4_)_2_SO_4_ (CAS 7783-20-2); 6 g KH_2_PO_4_ (CAS 7778-77-0); 0.2 g Na_2_HPO_4_ (CAS 7558-79-4); 1 mg FeSO_4_·7H_2_O (CAS 7782-63-0); 1 mg CaCl_2_ (CAS 10043-52-4); 10 µg H_3_BO_3_ (CAS 10043-35-3); 10 µg MnSO_4_ (CAS 10034-96-5)_;_ 70 µg ZnSO_4_ (CAS 7446-20-0); 50 µg CuSO_4_ (CAS 7758-98-7); 10 µg MoO_3_ (CAS 1313-27-5); 500 mg PCL (acetone:water); and 1 L distilled water [35].

The reagents used in the above formulations were obtained from Sigma Aldrich and Merck (Darmstadt, Germany). 

The results were scored as the diameter of the colonies in centimeters in both culture media, in addition to the formation of a whitening zone at the center of the colony in the medium with PCL.

### 2.2. PET Depolymerization Assays

The biodegradation tests were carried out by fermentation in a liquid medium with polymeric films of different brands: PET1 was a fragment of a 500 mL Crystal^®^ mineral water bottle, and PET2 was a fragment of a 2 L Sprite^®^ diet soda bottle. The bottles were purchased from local stores, and, after removing the bottlenecks, caps, and labels, fragments of approximately 4.5 cm × 2.5 cm (500 mg) were cut using sterile scissors and transferred to Petri dishes (adapted from Sharon and Sharon [36]; Malafatti-Picca et al. [18]). The thicknesses of the PET films were measured using a RoHS^®^ digital micrometer (Newton, NJ, USA), and thermal analyses of differential scanning calorimetry (DSC) were performed to determine the melting temperature and crystallinity percentage of the PET samples (Table 2). The differential scanning calorimetry (DSC) analyses to obtain the thermograms were performed using the Netzsch DSC 200 PC equipment (Waldkraiburg, Germany) with PET1 and PET2 polymers. Then, they were studied in the temperature range from 30 °C to 350 °C with a heating ramp of 10 °C·min^−1^ under a nitrogen flow (N_2_) of 50 mL·min^−1^ [37].

Figure S1 of the supplementary material contains the thermograms of the samples. Equation S1 of the supplementary material presents the data to obtain the crystallinity in percentage (%).

The fragments were weighed on an analytical balance (e = 0.001 g), washed with tap water and common neutral detergent, and rinsed with sterile distilled water. Afterward, they were immersed in 2% sodium hypochlorite for 1 h, rinsed again with sterile distilled water, and kept for 30 min under ultraviolet light inside the biological safety cabinet (492 watts of power at a 50 cm distance of PET fragments). The fragments were left in the drying oven at 40 °C until complete evaporation of the water.

The fungal strains were grown to produce fermented broth for the lipase and esterase activity. The fungal strains were grown for seven days at 28 °C in a BOD chamber (Biothec, Piracicaba, São Paulo, Brazil) and subcultured in test tubes containing PDA (20 g·L^−1^ glucose) with approximately 100 mg of 0.5 cm × 0.5 cm PET1 bottle fragments. A suspension of 10^6^ spores per mL or, in the case of non-sporulating fungi, part of the mycelial mass was aseptically transferred to 125 mL Erlenmeyer flasks containing 40 mL of liquid medium (Table 3) plus approximately 500 mg of previously cleaned PET fragments (as reported above).

The flasks were incubated at 28 °C and shaken at 150 rpm for fifteen or forty days. The content was transferred to sterile Falcon tubes (50 mL) and centrifuged at 4472× *g* and 8 °C. Then, the suspensions were filtered through a 0.45 µm cellulose membrane to separate the PET fragments from the mycelium present in the fermented liquid. Each assay was performed in triplicate with an abiotic control (only media and PET fragments, without fungi).

The post-fermentation *PET* fragments were subjected to a new disinfection process, as previously mentioned, until no mycelium could be seen (in the binocular stereomicroscope). Later, they were dried at 40 °C in a conventional drying oven until constant weight. The mass variation of the fragments was calculated based on Equation (2), where *BF* and *AF* represent the fragments before and after fermentation, respectively.

(1) $$Mass\;variation(\mathrm{mg})=\left(\frac{PET\;Mass\;BF-PET\;Mass\;AF}{PET\;Mass\;BF}\right)$$

(2) $$Mass\;variation\;\left(\%\right)=\left(\frac{mass\;PET\;AF-mass\;PET\;PF}{mass\;PET\;AF}\right)\times 100$$

### 2.3. Analysis of Post-Fermentation PET Fragments

#### 2.3.1. Attenuated total reflectance/Fourier transform infrared spectroscopy (FTIR/ATR) 

The PET1 and PET2 samples were characterized using infrared spectroscopy with a IRPrestige-21 spectrometer (Shimadzu, Chiyoda-ku, Tokyo, Japan) within the range from 4000 cm^−1^ to 400 cm^−1^ with 32 accumulations and a resolution of 2 cm^−1^.

#### 2.3.2. Scanning Electron Microscopy (SEM)

The PET fragments were covered with gold using a Sputter Coater SCD 050 (Bal-Tec, Balzers, Liechtenstein) apparatus at 25 milliamps for 40 s under a pressure of 80 Pa. The images were obtained using a TM 3000 Tabletop Microscope (Hitachi, Tokyo, Japan) with a voltage acceleration of 15 k (analytical mode from 1 Kv to 30 Kv) (adapted from Sepperumal, Markandan, Paljara [40]).

#### 2.3.3. Analysis of the Post-Fermentation Broth

##### Total Proteins

The total protein content of the fermented broth was determined using the Bradford method [41], and the concentrations were obtained through a standard curve of Bovine Serum Albumin solution, BSA-Sigma Aldrich (Saint Louis, MO, USA) with a linear range between 3.33 µg·mL^−1^ and 33.33 µg·mL^−1^ (R2 = 0.994, 6 calibrators, n = 3). The absorbance readings were performed on a spectrophotometer at a wavelength of 595 nm, corresponding to the anionic form of Coomassie Brilliant Blue G-250 (Bio-Rad, Mississauga, Ontario, CA, USA) when associated with proteins.

### 2.4. Enzymatic Activities

Lipase: The lipolytic activity was determined by the titration of fatty acids released by the hydrolysis of palm oil analytical grade, 76% (Supelco^®^, Bellefonte, PA, USA) [42]. Then, 1 g of the oil (lipid substrate), 4 mL of sterilized distilled water, and from 5 to 10 glass beads were transferred to an Erlenmeyer flask (125 mL) to then receive 1 mL of the mycelium-free fermented broth.

The flasks were incubated at 50 °C and shaken at 150 rpm for 30 min, and the reaction stopped by adding 10 mL of a solution of acetone:ethanol (1:1). The released fatty acids were quantified using titration with a standardized NaOH solution (0.05 mol·L^−1^) and by adding three drops of phenolphthalein (0.1% in absolute ethanol) as an indicator. A blank was prepared with 1 mL of distilled water. These assays were processed in triplicate and the enzyme activity was expressed in units of lipolytic activity (LA) corresponding to the number of enzymes capable of providing a micromol of fatty acids per min of reaction.

**Figure 2:**
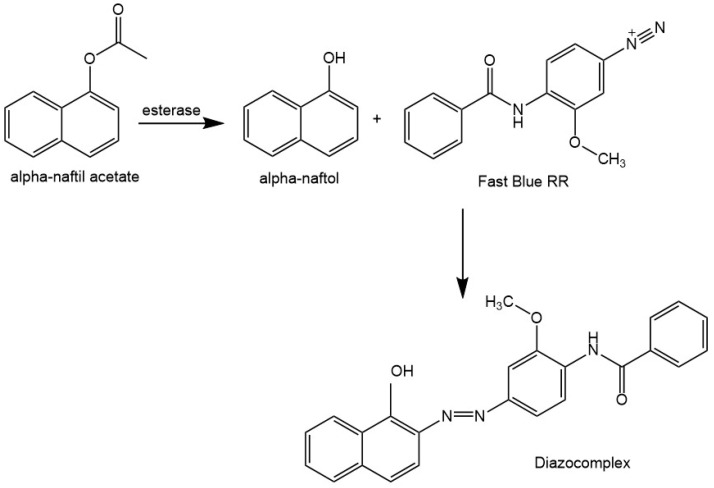
Diazo complex formation by esterase action on alpha-naphthyl acetate. Source: adapted from He [43].

Esterase: The esterase activity was estimated using the enzymatic hydrolysis of a 1.5 mmol·L^−1^ (alpha) α-naphthyl acetate solution (in a 300 mmol·L^−1^ phosphate buffer containing isopropyl alcohol). The aim was the quantification of α-naphthol, revealed by the formation of a diazo-complex after adding Fast Blue RR (0.1 mg·mL^−1^ in dimethyl sulfoxide and ethanol, in a relation of 1:2) (Sigma-Aldrich, Saint Louis, MO, United States) (Figure 2). The quantification was determined against an α-naphthol calibration curve at concentrations from 10 µmol·L^−1^ to 100 µmol·L^−1^ (5 calibrators, n = 3, R^2^ = 0.999). Spectrophotometric readings were performed using the 490 nm wavelength [43]. The samples were analyzed using test tubes, and 100 µL of the fermented broth was added to 2 mL of α-naphthyl acetate, then incubated in a water bath at 37 °C for 1 h. The reaction was interrupted by adding 1.0 mL of sodium dodecyl sulfate (10% in sterile water, pH corrected to 7.0); 1.0 mL of Fast Blue RR was added to reveal the α-naphthol formed, and the absorbances were determined against a blank containing 100 µL of sterile water.

### 2.5. Terephthalic Acid (PTA) and Oligomers (BHET and MHET) Using HPLC-UV

**Figure 3:**
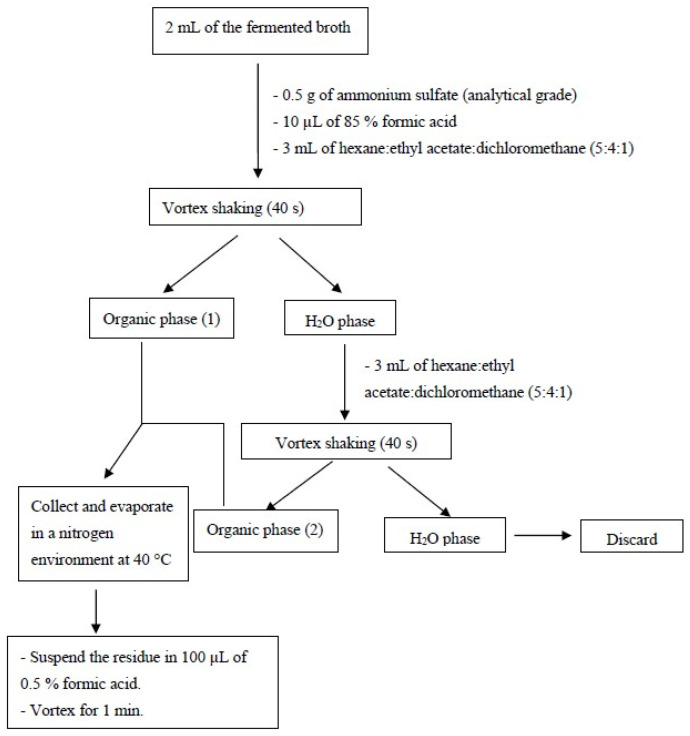
Flowchart of the liquid–liquid extraction for PTA, BHET, and MHET purification and pre-concentration from the fermented broth (adapted from Kim, Lee [44]).

**Figure 4:**
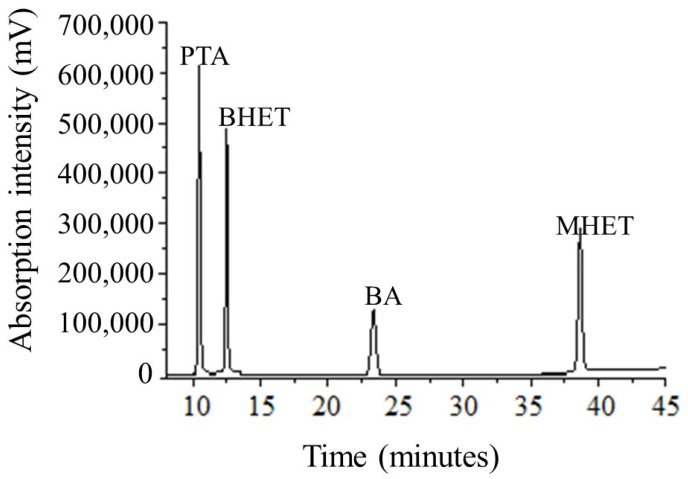
Chromatogram of terephthalic acid (PTA), bis(2-hydroxyethyl) terephthalate (BHET), benzoic acid (BA), and 2-hydroxyethyl methyl terephthalate (MHET) analytes (standard solutions 240 ppm, injection volume = 3 µL).

The PTA, BHET, and MHET released in the fermented broth were obtained using liquid–liquid extraction (adapted from Kim and Lee [44]) (Figure 3). The chromatographic separations were performed on a Shimadzu equipped with an octadecylsilane (C18) column Shim-pack VP-ODS (4.6 mm × 250 mm, 5 µm) coupled to a pre-column of the same material (Shimadzu, Santa Clara, CA, USA), with a manual injector and using the following mobile phases: Solvent A: 0.5% (*v*/*v*) formic acid:acetonitrile, HPLC grade 90:10, and Solvent B: 0.5% (*v*/*v*) formic acid:acetonitrile, HPLC grade 60:40. These mobile phases were followed by an elution gradient, according to Table S1 (supplementary material), in a flow of 0.5 mL·min^−1^. The analytes were detected at a wavelength of 254 nm. Benzoic acid (retention time = 23.4 min) was used as an internal standard. The method was the following: linearity from 0.15 ppm to 300.0 ppm for PTA, BHET, and MHET, with a correlation coefficient of 0.999 for the three analytes (6 calibration solutions n = 3); precision determined by the relative standard deviation (variation coefficient) between 1.1% and 31.1%. The latter was the lowest concentration of the curve. The chromatogram in Figure 4 shows the separation efficiency of the methodology used.

### 2.6. Fungal Identification

The genomic fungal DNA was extracted following the modified method by Aamir et al. [45] using phenol, chloroform, and isopropyl alcohol. The regions translation elongation factor-1α (tef1) (for *Trichoderma* spp.) and ITS (for other genera) were amplified. The primers and amplification programs are shown in Table 4.

The polymerase chain reactions (PCRs) for the ITS region were performed with a final volume of 25 µL containing 0.2 µL of dNTPs (25 mmol·L^−1^, Invitrogen, Waltham, MA USA), 2.5 µL of Taq buffer (10x-Invitrogen), 1.5 µL of MgCl_2_ (50 mmol·L^−1^, Invitrogen), 0.5 µL of each primer (20 mmol·L^−1^, Invitrogen), 0.2 µL of Taq polymerase (5 U·µL^−1^), 3.45 µL of diluted genomic DNA (15 ng·µL^−1^), and 16.15 µL of sterile ultrapure water. The reagent concentrations in the final solution were the following: 0.2 mmol·L^−1^, 3 mmol·L^−1^, 0.4 mmol·L^−1^, 0.04 U·µL^−1^, and 2.07 ng·µL^−1^ for dNTPs, primer, MgCl_2_, Taq polymerase, and genomic DNA, respectively.

The PCR reactions for the Tef region had the same final volume with 0.2 µL of dNTPs (25 mmol·L^−1^, Invitrogen) plus 2.5 µL of Taq buffer (10x-invitrogen), 1.5 µL of MgCl_2_ (50 mmol·L^−1^, Invitrogen), 1.0 µL of each primer (10 mmol·L^−1^), Bovine Serum Albumin—BSA (1 mg·mL^−1^, Promega), 0.2 µL of Taq polymerase (5 U µL^−1^, Invitrogen), 3.45 µL of diluted genomic DNA (15 ng.µL^−1^), and 14.15 µL of sterile ultrapure water.

The amplicons were purified using the Illustra GFX™ PCR DNA and Gel Band Purification Kit (GE Healthcare, Buckinghamshire, UK) and subjected to the sequencing reaction using the same primers with the following program: 95 °C for 1 min followed by 28 cycles at 95 °C for 15 s, 50 °C for 45 s, and 60 °C. After the reaction, all the samples were removed for further purification and sequenced with the BigDye Terminator (Life Technologies, Waltham, MA USA) in an ABI3500XL series sequencer (Applied Biosystems, Waltham, MA USA) according to the manufacturer instructions.

The BioEdit Sequence Alignment Editor v.7.0.5.3 [48] was used to generate the consensus sequence, which was compared with sequences from organisms represented in the GenBank nucleotide database (National Center for Biotechnology Information (nih.gov) (accessed on 10 February 2023) and CBS database (Westerdijk Institute (knaw.nl)) (accessed on 10 February 2023). The sequences of organisms related to the unknown fungi were selected and used for further genetic distance analysis. After the alignment using the CLUSTAL X software [49], a phylogenetic tree based on genetic distance was generated using MEGA software version 4.0 [50] with the neighbor-joining method [51] and bootstrap values calculated from 1000 replicates. The sequences of the genetic material of the fungi highlighted in this work were deposited in the GenBank receiving the following accession numbers: *Curvularia trifolii* CBMAI 2111 (OL804101); *Trichoderma atroviride* CBMAI 2073 (OM275355); and *Cladosporium cladosporioides* CBMAI 2075 (OL814981) and *Trichoderma* sp. CBMAI 2071 (OM275356).

### 2.7. Statistical Analyses

The data assumed a non-parametric distribution and were analyzed using the Kruskal–Wallis test, followed by mean comparison studies with the Student–Newman–Keuls test. The analyses were performed using the Bioestat 5.3^®^ software (Belém, PA, Brazil), considering a 95% confidence interval, *p* ≤ 0.05.

## 3. Results

### 3.1. Enzymatic Assays with PET Nanoparticles

From a total of 100 fungal isolates, 26 produced extracellular enzymes with the potential to convert the polymer nanoparticles into terephthalic acid. Of these, five strains exhibited conversion percentages of 2% or higher (Table 5). Table 5 shows the lineages (in bold) that are part of this study. The selected strains presented good results in the tests with PET nanoparticles, with a considerable mycelial aggregation to the PET polymer of bottles during the biodegradation tests.

### 3.2. Cutinase Production

**Figure 5:**
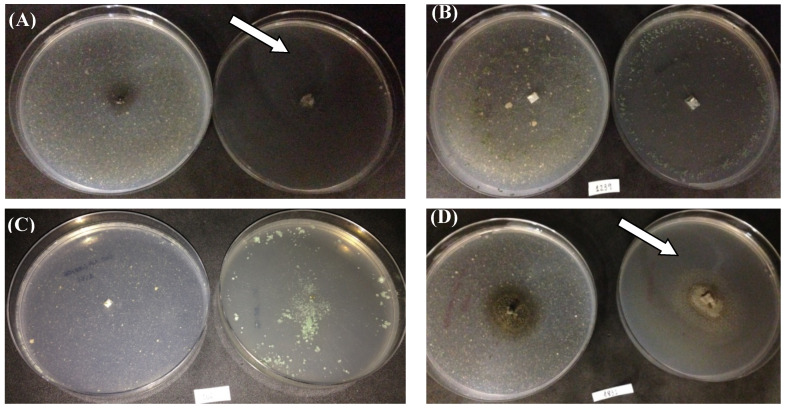
Qualitative assay for probing cutinase production using promising strains. Note. (**A**). *C. trifolii* CBMAI 2111; (**B**). *T. atroviride* CBMAI 2073; (**C**). *T.* sp. CBMAI 2071; (**D**). *C. cladosporioides* CBMAI 2075. Plates with minimal mineral media containing cutin (left) and polycaprolactone (right) as the sole carbon sources. White arrows indicate the consumption of polycaprolactone and cutin polymer by fungi, in addition to microbial growth.

Figure 5 shows the growth of isolates capable of assimilating cutin or polycaprolactone as the sole carbon sources. *Curvularia trifolii* CBMAI 2111 (A) and *Cladosporium cladosporioides* CBMAI 2075 (D) exhibited colonies with 1.5 cm and 1.8 cm diameters in the presence of cutin and 3.5 cm and 4.5 cm for the medium with PCL, respectively. In addition, the decolorization zones were observed in the culture plate with PCL, indicating polymer degradation (see white arrows). Figure S2 of the supplementary material shows overlays of FTIR images of the cutin and PCL.

### 3.3. PET Depolymerization Assays

**Figure 6:**
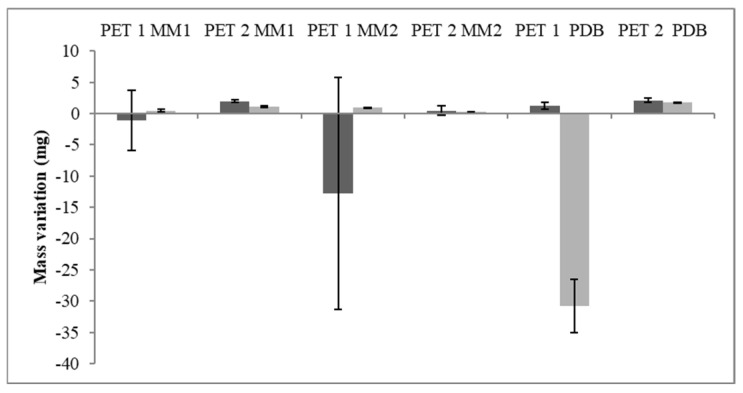
Mass variation of the PET fragments after fermentation. *C. cladosporioides* CBMAI 2075 (light gray) and *T. atroviride* CBMAI 2073 (dark gray). Data below the *x*-axis (negative values) indicate mass gain at the end of the process.

Due to the growth in cutin and polycaprolactone media and with npPET studies, the strains *T. atroviride* CBMAI 2073 and *C. cladosporioides* CBMAI 2075 were chosen for further tests with culture media containing different rates of glucose and palm oil as the sole carbon source. After 40 days of fermentation, a tendency for a mass gain of the PET1 fragments was observed, while the PET2 fragments lost mass in the three tested culture media (Figure 6). MM1 and PDB showed a greater reduction in the mass of the PET2 polymers for both isolates studied, showing a variation between 1.2 mg and 2.2 mg.

**Figure 7:**
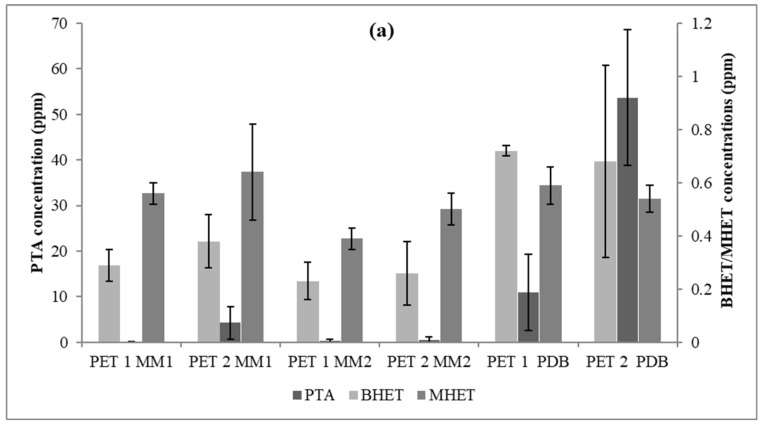

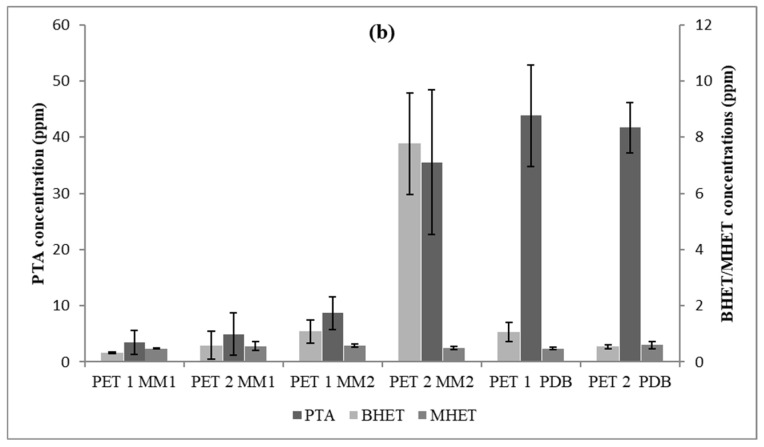
PTA, BHET, and MHET concentrations released from PET1 and PET2 with three different culture media (time = 40 days). (**a**) *Trichoderma* sp. CBMAI 2073; and (**b**) *Cladosporium* sp. CBMAI 2075.

The enzymatic activities were evaluated in the fermented broth (Table 6). *T. atroviride* (CBMAI 2073) grown in MM2 showed higher esterase and lipolytic activity on PET1 and PET2 fragments, respectively. *C. cladosporioides* CBMAI 2075 had more evident esterase activity in MM1 and PDB (on PET1), while a higher lipase value was found in MM2 (on PET2). The activity determined showed high standard deviation, which could be attributed to the variability in the microorganism growth and enzyme production. Both strains were able to depolymerize PET in by-products such as terephthalic acid, bis(2-hydroxyethyl) terephthalate, and 2-hydroxyethyl methyl terephthalate (Figure 7).

The statistical analyses suggested that in the PDB medium, CBMAI 2073 had a greater release of the PTA monomer on both PET types (*p* < 0.05). It was also observed that BHET was available in a greater amount in PDB (*p* < 0.05) using PET1, unlike the result for MHET, which did not show any significant difference regarding the treatment applied to both types of polymers (*p* > 0.05).

**Figure 8:**
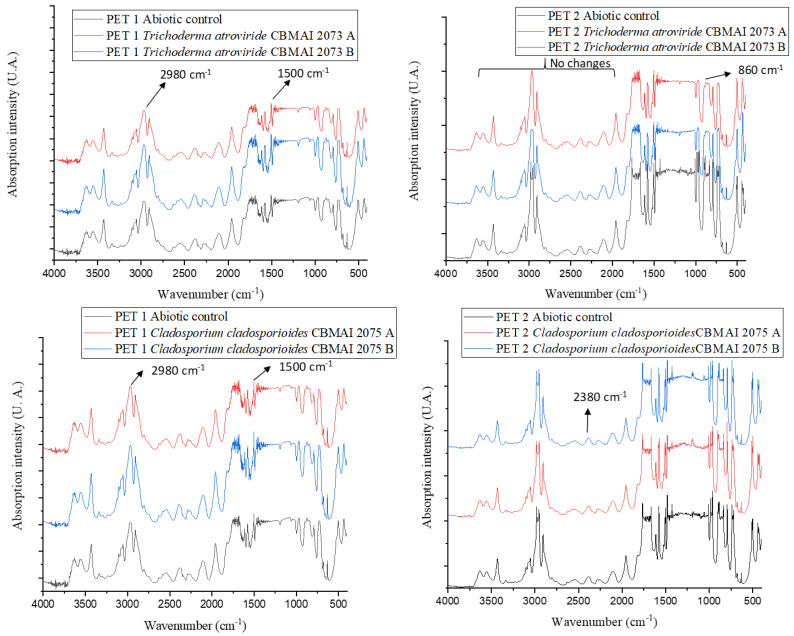
FTIR spectra of PET1 and PET2 polymers after 40 days in contact with *T. atroviride* CBMAI 2073 and *C. cladosporioides* CBMAI 2075. Note. Culture media PDB, t = 28 °C, shaking at 150 rpm. The FTIR analysis was performed in duplicate treatments, indicated as A and B in the figure.

Figure 8 presents the infrared spectra for PET1 and PET2. In general, CBMAI 2073 and CBMAI 2075 revealed a greater number of molecular changes for PET2, as seen in Table 7. The disappearance of bands was observed in the PET, suggesting a modification of the chemical structure. The disappearance of bands was noted at the region of 983 cm^−1^, 962 cm^−1^, and 835 cm^−1^, which could be related to the aromatic ring. The bands at the region of 1481 and 1427 cm^−1^ could be related to stretching of the C-O group, and deformation of the O-H group. The vibration of the aromatic skeleton (C=C) could be attributed to the band at 1579 cm^−1^.

Fewer modifications were observed with PET1 (Table 7). The fermentation of CBMAI 2073 showed an increase in band intensity at 1579 cm^−1^ and 1573 cm^−1^, band displacement from 630 cm^−1^ to 632 cm^−1^, band appearance at 613 cm^−1^, and band disappearance at 656 cm^−1^. The only change with CBMAI 2075 was the increase in the band intensity at 1579 cm^−1^ and 1573 cm^−1^.

**Figure 9:**
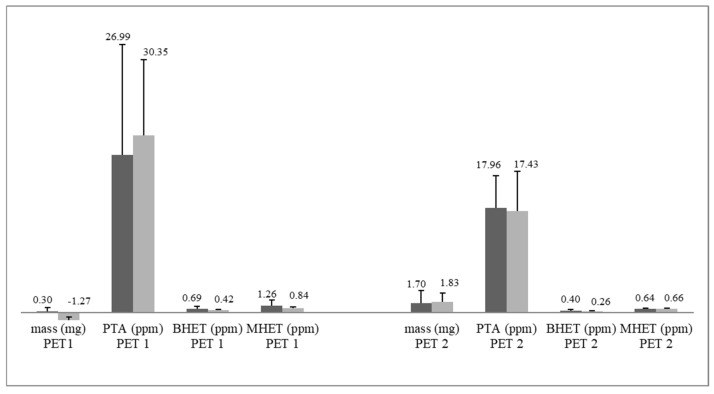
Mass variation (mg) and PTA, BHET, and MHET concentrations released in the fermented broth of *C. trifolii* (CBMAI 2111) (color dark grey) and *T.* sp. (CBMAI 2071) (color light grey). Fermentation time = 15 days, t = 28 °C, and 150 rpm. (Data expressed as mean + standard deviation, n = 3. Values below the *x*-axis indicate the mass gain of the fragment).

Figure S3A–P in the supplementary material contains the FTIR spectra in the zoomed view for the best observation of the alterations promoted by the treatments. Based on these results, PDB was selected for the depolymerization tests with two more strains: *Curvularia trifolii* CBMAI 2111 and *Trichoderma* sp. CBMAI 2071. Both strains were selected because they indicated the degradation of the polymers PCL and cutin by their growth rates. Thus, 15-day fermentations were carried out. Figure 9 shows the variation data of the PET masses, as well as the concentrations of monomers and oligomers released by the enzymatic action.

The data in Table 8 indicate that extracellular hydrolases were produced by the strains studied in the presence of PET fragments.

**Figure 10:**
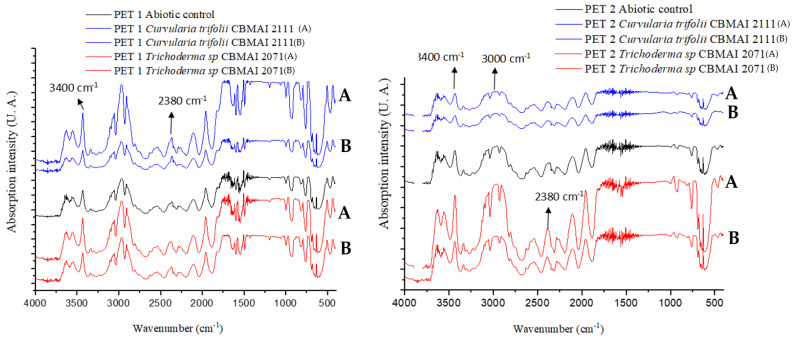
FTIR spectra for PET1 and PET2 obtained after 15 days in contact with *C. trifolii* CBMAI 2111 and *Trichoderma* sp. CBMAI 2071. Culture media PDB, t = 28 °C, 150 rpm. The FTIR analysis was performed in duplicate treatments, indicated as A and B in the figure.

Compared to the abiotic control, the infrared spectra of PET1 and PET2 fragments (Figure 10) treated with *C. trifolii* CBMAI 2111 and *T*. sp. CBMAI 2071 showed the changes highlighted in Table 9.

Figure S4A–H in the supplementary material contains the FTIR spectra in the zoomed view for the best observation of the alterations promoted by the treatments.

**Figure 11:**
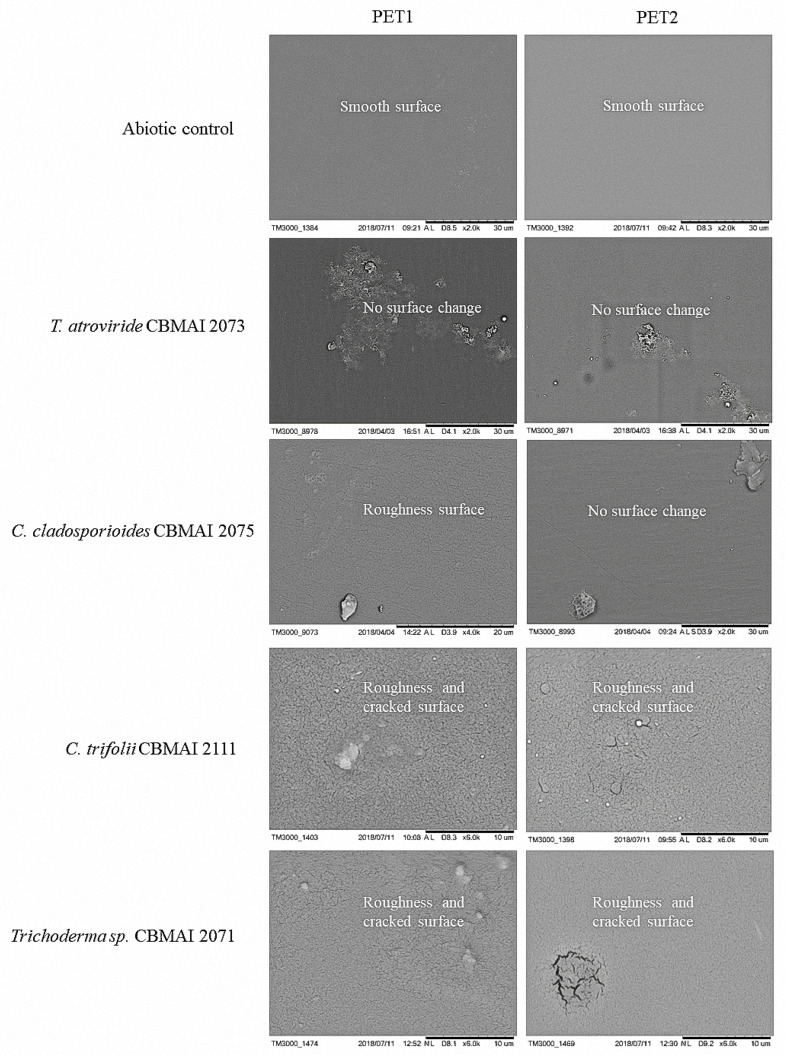
Scanning Electron Microscopy (SEM) demonstrating the effect of *T. atroviride* CBMAI 2073, *C. cladosporioides* CBMAI 2075, *C. trifolii* CBMAI 2111, and *T.* sp. CBMAI 2071 on PET1 and PET2 fragments compared to the abiotic control (PDB medium, no inoculum). Note. The images were obtained after cleaning the PET fragments after 40 days in PBD, 28 °C, and 150 rpm.

Figure 11 shows the SEM micrographs for the PET1 and PET2 fragments after fermentation in PDB media, highlighting the effect on the polymers for all treatments.

## 4. Discussion

A total of 26 strains showed the potential to enzymatically hydrolyze PET nanoparticles. The strains with the ability to depolymerize are known to be good producers of hydrolases such as lipases, esterases, and cutinases [52].

The preliminary use of npPET to test the enzymatic activity was addressed to select the representatives that produce polyester hydrolases. In general, nanoparticles have a high ratio of exposed surface by volume occupied, which allows for greater enzymatic access compared to other structural forms such as films or fibers [53]. Using the high-throughput screening test (HTS), six strains with values above 2% of npPET conversion were identified, while the best results in previous works were limited to 1%, except for a strain of *Trichoderma* sp. L1239 with 7.1% [30].

In this screening, four isolates were selected for further analysis: *Curvularia trifolii* CBMAI 2111, *Trichoderma* sp. CBMAI 2071, *Trichoderma atroviride* CBMAI 2073, and *Cladosporium cladosporioides* CBMAI 2075 (Table 2). The selection was based on the best results of the NPPET analysis and for presenting strong mycelial adhesion to the PET-bottle polymer, as per the preliminary tests reported for strains *Microsphaeropsis arundinis* CBMAI 2109 and CBMAI 2110 [18].

The evaluation of the enzymatic activities of the selected strains on short (two carbons) and long (eight carbons) esterified fluorescent probes was previously performed (Table S1). In general, it was observed that the strains did not show good results for this former type of tested probe (two and eight carbons) with conversion rates below 50% according to the criteria established by Malafatti-Picca et al. [18]. The strain *T.* sp. CBMAI 2071, for example, was not able to convert the probes under the conditions tested (Table S1), showing no conversion for probes (substrates) with two and eight carbons. However, these data corroborate those found by Yoshida et al. (2016) [21], who identified a new bacterial species, *Ideonella sakaiensis*, capable of adhering to the PET surface and secreting enzymes characterized as cutinase-like (PETases), which assimilated the PET polymer in preference to other substrates.

Plate tests with minimal mineral media containing cutin and polycaprolactone (MM 10,000 g·mol^−1^) were performed with the selected isolates (Figure 6) and revealed their ability to assimilate both polymers. Nimchua et al. [19] used a minimal medium with PCL in the isolation of enzyme-producing filamentous fungi, which increased the hydrophilicity of PET fibers. Polycaprolactone is considered an analog of cutin, an insoluble biopolyester and structural component of plant cuticles [35]. Figure S2 of the supplementary material overlays the FTIR spectra of these polymers and indicates similarity between the two compounds.

The genera *Curvularia* [54], *Cladosporium* [55], and *Trichoderma* [56] contain plant pathogen species but also others that benefit the growth of many plants. They are ubiquitous with representatives that are considered “indoor fungi”, and well-known for producing different enzymes [57]. The literature reports the presence of genes encoding cutinase in the genome of representatives of *Curvularia lunata, Trichoderma reesei*, and *Cladosporium fulvum* [58,59,60]. The results obtained herein by the qualitative test in plates were consistent and justified the affinity of these enzymes with the NPPET substrate and their low affinity with the esterified fluorogenic probes, which agrees with the data reported by Yoshida et al. [21]. However, it is important to highlight that a more in-depth investigation of the type of enzyme produced by the selected strains is recommended.

Although the nanoparticles are considered structurally favorable to the enzymatic action when aiming at the depolymerization of PET, they do not correspond to the final disposition of the polymer. The transformation of all PET to be recycled into nanoparticles to undergo enzymatic hydrolysis could also be a good alternative; however, this process may use halogenated or metallic organic solvents that are miscible in water and unsafe for the environment [61]. Therefore, further tests were carried out with the most promising strains on PET bottle fragments, which is the structure most found in different environments.

The present investigation used two types of polymers denominated PET1 and PET2. The former comes from the “plant bottle” technology and uses 30% of PET derived from sugar cane, referring to mono-ethylene glycol (MEG) and called BioMEG [62]. The second uses conventional technology based on non-renewable fossil resources as precursors. Table 2 shows the different crystallinity percentages determined using DSC analyses: 35.47% and 10.41% (PET1 and PET2, respectively). Wei and Zimmerman [63] reported that PET from bottles and fibers with a high degree of crystallinity (above 30%) represent the most abundant type of post-consumer polymer and may not be readily hydrolyzed efficiently in enzymatic processes. The “plant bottle” technology manufacturer reports that the biodegradation of the polymer does not differ from conventional technologies; however, some studies showed greater enzymatic degradation efficiency for polymers with crystallinity of around 10% [24,64].

The strains *T. atroviride* CBMAI 2073 and *C. cladosporioides* CBMAI 2075 were chosen to test the culture medium that best favored PET depolymerization. Among the media tested (MM1, MM2, and PDB—Table 3), both strains expressed better activities in PDB, promoting the depolymerization of PET into PTA and BHET with a significant difference at the 95% confidence level (*p* < 0.05). Both the minimal mineral medium [65,66,67] and those rich in glucose [68] have been used in polymer biodegradation or depolymerization studies. In this study, the parameters used to evaluate the polymer degradation were mass variation, FTIR spectra, and scanning electron microscopy images. In the fermented broth, concentrations of monomers (PTA) and oligomers (BHET and MHET) were measured, as well as enzymatic activities.

As most studies on biodegradation apply mass variation to assess the effect of strains on the polymer [36,69,70], it was also included here, but the results were considered inconsistent (Figure 7 and Figure 10). El-Saffei et al. [71] also considered this approach inconclusive as mass gain may occur either by the adhesion of the mycelia to the fragment or the oxidation or water absorption of the culture medium.

The PET recalcitrance characteristics discourage depolymerization studies, causing little information to be available in the literature on polymer analyses using FTIR. According to Holland and Hay [72] and Vijayakumar and Rajakumar [73], the spectra from bottles must contain some vibrations from groups that confirm the presence of important chemical bonds functioning as “fingerprints” for that material. They may be 1713 cm^−1^ (presence of carbonyl group C=O conjugated to the aromatic ring), 1234 cm^−1^ (asymmetric C-C-O stretching involving carbon in the aromatic ring), 728 cm^−1^ (aromatic ring C-H movement outside the plane), 872 cm^−1^ (C-H stretching of the aromatic ring outside the plane), 1128 cm^−1^ and 1091 cm^−1^ (O-C-C split asymmetric stretching), 2960 cm^−1^ (asymmetric stretching of C-H), 1505 cm^−1^ (aromatic ring C-C stretching), 1453 cm^−1^ (deviation/folding of C-H), and bands at 1408 cm^−1^ and 1339 cm^−1^ (C-H deformation of the alkane). The vibrations, stretching, and folding may vary from 0.5 cm^−1^ to 1.5 cm^−1^ on average. The treatments with *C. trifolii* CBMAI 2111, *T.* sp. CBMAI 2071, *T. atroviride* CBMAI 2073, and *C. cladosporioides* CBMAI 2075 led to changes at various points of the spectrum in the regions mentioned above (Figure 9 and Figure 11). Analyzing *Penicillium* spp. and PET, Nowak et al. [70] and Yamada-Onodera et al. [74] obtained similar results to those found in this work. After studying the biodegradation of polyethylene, Jumaah [75] concluded that some modifications observed using FTIR indicate the microbial action on the recalcitrant element. This is considered a good result because it reveals that a strain capable of promoting such modifications (albeit small) may also synergistically interact with others, approaching the mineralization of the compound. It was clear that the FTIR spectra of PET2 in treatments with the most promising strains (*C. trifolii* CBMAI 2111, *T.* sp. CBMAI 2071, *T. atroviride* CBMAI 2073, and *C. cladosporioides* CBMAI 2075) were more likely to have molecular changes than PET1 samples, thus showing that they are better degraded by the strains. The results from Table 4 highlight the high crystallinity of PET1, which would proportionally have a smaller amount of the polymer in its amorphous form and is preferred for the enzymatic action of hydrolases [76].

The enzymatic activities quantified after the fermentation of PET1 and PET2 with all strains studied (Table 6 and Table 8) proved the production of extracellular enzymes (lipases and esterases), which could act on the polymer. The wide use of *Trichoderma* strains to produce industrial biomolecules is well-known and scientifically reported as one of the most promising genera for biotechnology. The better lipase and esterase activities observed in the fermented broth of CBMAI 2071 was expected (Table 8). On the contrary, the good performance of CBMAI 2111 (*Curvularia trifolii*) was considered a novelty for this species. According to Yamada-Onodera [74], extracellular biological catalysts produced by hyphae may help in the rapid degradation of polymers. Hiraga et al. [23] consider it is important to elucidate the mechanisms of cell adhesion to polymers, as it is certainly what could implement the PET biodegradation process.

To confirm the PET depolymerization, the determination of monomers and oligomers is the most important among the evaluated parameters since they are the by-products that will originate new bottles. This approach meets the needs of the current recycling context and allows the selection of strains with greater effectiveness in the depolymerization of this material.

The quantification of monomers and oligomers is a difficult task as it may involve a slow process such as the assimilation of a polymer by the microorganism [77]. The previous experiments carried out using chromatographic determinations of PTA in fermented broth without pre-concentration led to quantification data below the established limit for chromatographic separation under the conditions established for this work. Pre-concentration strategies such as liquid-liquid extraction are often necessary to increase analyte recovery and result in reliability [44].

In this research, the chromatographic separation of the PTA, BHET, and MHET analytes was achieved after liquid–liquid extraction by reverse-phase high-performance liquid chromatography. The results showed that the strains under study were able to convert the PET1 and PET2 fragments into concentrations of terephthalic acid monomer (PTA) between 12 ppm and 45 ppm (for PET1) and between 17 ppm and 55 ppm (for PET2). Considering the visible increase in microbial biomass, it is possible that the fungi tested may have assimilated the mobilized compounds (PTA, BHET, and MHET) as reported by Gong et al. [78], thus the concentration of monomers and oligomers released from PET were possibly higher.

Some previous works that evaluated PET biodegradation/depolymerization often used microbial enzymes and the biocatalysis promoted by isolated enzymes. The process was improved by applying the polymer with different physical characteristics (amorphous PET and low-crystallinity product). Zhang et al. [79] demonstrated the degradation of PET fibers mediated by unidentified microorganisms (synergism between bacteria and fungi) with conversions into terephthalic acid at concentrations between 0.3 ppm and 3.0 ppm. Danso et al. [13] evaluated the biodegradation potential of an enzyme from the marine environment using a metagenomic approach (called PET 2 in their work) on 14 mg of amorphous PET and obtained 900 µM (149 ppm) of terephthalic acid after 24 h. Another study used isolated cutinases produced by *Thermobifida fusca* (TfCut2) (Actinobacteria) and recombinant cutinases (TfCut2 G62A/F209A) designed based on the cutinase gene sequence of *Ideonella sakaiensis*. In the presence of a cationic surfactant, the cutinases degraded low-crystallinity (3% to 5%) PET with 200 μm thickness and PET with high crystallinity (40%) and 190 μm thickness. The polymer studied was obtained commercially, corresponding to a very homogeneous material [80]. The strains studied in this work are considered highly promising for PET depolymerization when the results are compared with those reported in the literature. The highlights of this study may be listed as follows: 1. the application of microbial strains as whole cells under room temperature (approximately 28 °C); 2. PET samples from commercial bottles, thus corresponding to heterogeneous material with different crystallinities; and 3. there are no reports of PET biodegradation capacity for the fungal species identified here except for *C. cladosporioides* [25], which highlights the novelty of the data obtained.

A hypothesis for the promising result of the *Trichoderma atroviride* strain CBMAI 2073 is based on Espino-Hammer et al. [81], who reported that some species of *Trichoderma* can produce class II hydrophobin molecules that stimulate the activity of cutinases on PET.

Although analysis using scanning electron microscopy (SEM) is usually considered only qualitative to evaluate the effects of microbial growth on polymers [82], its use was considered highly valuable here. The images obtained (Figure 11) clearly indicated that the four selected strains were able to promote structural changes on the material and corroborated the results obtained in the other analyses carried out in the present investigation.

The work with the selected strains enabled us to gain insights into important applications in PET depolymerization. It is recommended that further studies focus on the isolation, identification, and use of these enzymes in biodegradation assays, in addition to promoting synergistic interactions between the isolates to try to optimize this process.

## 5. Conclusions

Considering that this work aimed to elucidate important points, better guide PET biodegradation studies, which are scarce, and highlight the fungal strains with the potential to assimilate the polymer, the contributions are as follows: between the two types of PET addressed in this study, the material with the highest crystallinity (here called PET1) showed greater resistance to enzymatic action. *Trichoderma* sp. CBMAI 2071, *Trichoderma atroviride* CBMAI 2073, *Cladosporium cladosporioides* CBMAI 2075, and *Curvularia trifolii* CBMAI 2111 are possible cutinase-producers because they can grow in a culture medium with cutin and polycaprolactone as the sole carbon sources, according to qualitative analyses. Compared to other culture media, Potato Dextrose Broth (PDB) favored the conversion of Polyethylene Terephthalate into PTA, BHET, and MHET during fermentation with the strains *Trichoderma* sp. CBMAI 2071 and *C. cladosporioides* CBMAI 2075. The studied strains *Trichoderma* sp. CBMAI 2071, *Trichoderma atroviride* CBMAI 2073, *Cladosporium cladosporioides* CBMAI 2075, and *Curvularia trifolii* CBMAI 2111 showed action on depolymerization according to the analysis of the enzymatic activities and the release of monomers (concentrations above 12 ppm) and oligomers (concentrations above 0.6 ppm) in the fermented liquid. They are also capable of promoting important changes in the chemical structure and surface of the polymer (according to FTIR and SEM). The evaluation of mass variation, however, seems to be an inconclusive parameter of inconstant results.

## Figures and Tables

**Table 1:** Components per assay for analyses with PET nanoparticles.

Assay	Fungal Cells (Suspension, 1 mg·mL^−1^, in 20 mM Borate Buffer—pH 7.8)	PET Nanoparticles * (Suspension, 0.11 mg·mL^−1^)	Terephthalic Acid (Solution, 2.6 mM)	Borate Buffer (20 mM—pH 7.8)
Enzymatic	100 µL	30 µL	--	---
Positive control	100 µL	---	100 µL	---
Negative control	---	30 µL	100 µL	---
Microbial control	100 µL	---	---	30 µL

* Diameter of approximately 60 nm [30,31].

**Table 2:** Physical characteristics obtained using DSC analysis for the PET1 and PET2 samples.

Sample	Thickness (mm)	T_m_(°C)	ΔH_m_ (J·g^−1^)	X (%)
PET1	0.10 ± 0.01	251.12	49.66	35.47
PET2	0.21 ± 0.01	253.41	14.57	10.41

T_m_°C = melting temperature; ΔH_m_ (J·g^−1^) = melting enthalpy; X (%) = crystallinity percentage. Reference: PET 100% Crystalline ΔHm = 140 J·g^−1^ [38].

**Table 3:** Culture media used in the PET depolymerization test.

Liquid Culture Media *	Components and Amounts in g·L^−1^	
Minimal mineral media (MM1)	KH_2_PO_4_	1.0
	KNO_3_	1.0
	MgSO_4_	0.5
	KCl	0.5
	Yeast extract	0.2
	Sucrose	0.2
	Glucose	0.2
	Distilled water	1 L
Minimal mineral medium supplemented with palm oil (MM2)	KH_2_PO_4_	1.0
	KNO_3_	1.0
	MgSO_4_	0.5
	KCl	0.5
	Yeast extract	0.2
	Palm oil	0.4
	Distilled water	1 L
Sigma-Aldrich^®^ Potato dextrose broth (PDB)	Glucose (Dextrose)	20
	Potato infusion	4.0
	Distilled water	1 L

* Culture media was based on published material elsewhere [39].

**Table 4:** Primers and amplification conditions.

Region	Primer	Sequence (5′–3′)	Amplification Program	References
**ITS**	ITS1 (forward)	TCCGTAGGTGAACCTGCGG	Initial denaturation at 94 °C for 2 min, 30 denaturation cycles at 94 °C for 1 min, annealing at 55 °C for 1 min, extension at 72 °C for 3 min, and final extension step at 72 °C for 3 min and 4 °C.	[46]
	ITS4 (reverse)	TCCTCCGCTTATTGATATGC		
**Elongation factor 1α (*tef1*)**	EF1-728F (forward)	CATCGAGAAGTTCGAGAAGG	Initial denaturation at 94 °C for 2 min, 15 denaturation cycles at 94 °C for 30 s, annealing at 65 °C for 30 s, extension at 72 °C for 1 min, followed by 35 cycles at 94 °C for 30 s, 48 °C for 30 s, and final extension step at 72 °C for 1 min.	[47]
	TEF1R (reverse)	GCCATCCTTGGAGATACCAGC		

**Table 5:** PET nanoparticle conversion (%), results after fifteen days of incubation.

Code	Identification	npPET Conversion (%) *	Code	Identification	npPET Conversion (%) *
**CBMAI 2111**	*Curvularia trifolii*	**9.0 ± 1.1**	**CBMAI 2203**	*Paraconiothyrium cyclothyrioides*	0.6 ± 0.3
**CBMAI 2073**	*Trichoderma atroviride*	**6.1 ± 0.2**	**LMA 216**	*Phoma herbarum*	0.6 ± 0.6
**CBMAI 2071**	*Trichoderma* sp.	**3.6 ± 0.8**	**LMA 28**	*Aspergillus fumigatus*	0.5 ± 0.0
**CBMAI 2110**	*Microsphaeropsis arundinis*	**2.7 ± 0.9**	**LMA 1825**	*Fusarium* sp.	0.4 ± 0.0
**CBMAI 2109**	*Microsphaeropsis arundinis*	2.0 ± 0.4	**LMA 1172**	*Fusarium* sp.	0.4 ± 0.0
**LMA 1269**	*Fusarium* sp.	1.9 ± 0.5	**CBMAI 2190**	*Aspergillus fumigatus*	0.4 ± 0.2
**CBMAI 2159**	*Pseudallescheria* sp. *(Complexo Pseudallescheria*/*Scedosporium)*	1.9 ± 1.0	**CBMAI 2186**	*Talaromyces veerkampii*	0.3 ± 0.2
**CBMAI 2083**	*Paecylomyces* sp.	1.7 ± 0.7	**CBMAI 2187**	*Paraconiothyrium cyclothyrioides*	0.3 ± 0.3
**CBMAI 2189**	*Aspergillus fumigatus*	0.8 ± 0.6	**CBMAI 2149**	*Trichoderma capillare*	0.3 ± 0.0
**CBMAI 2075**	*Cladosporium cladosporioides*	**0.8 ± 0.6**	**CBMAI 2155**	*Microsphaeropsis arundinis*	0.3 ± 0.2
**LMA1251**	*Trichoderma* sp.	0.7 ± 0.4	**LMA 167**	*Penicillium* sp.	0.3 ± 0.1
**CBMAI 2158**	*Paraconiothyrium cyclothyrioides*	0.7 ± 0.1	**LMA 11**	*Paecilomyces*-like	0.2 ± 0.0
**CBMAI 2191**	*Penicillium koreense*	0.7 ± 0.1	**LMA 1145**	*Fusarium* sp.	0.1 ± 0.0

* Conversion results from NPPET into HOTP expressed as mean percentage ± standard deviation (n = 3).

**Table 6:** Lipase and esterase activities in the fermented broth.

*T. atroviride* CBMAI 2073	PET 1			PET 2		
	MM1	MM2	PDB	MM1	MM2	PDB
Lipase (UA)	0.69 ± 0.0	0.64 ± 0.2	0.47 ± 0.1	0.58 ± 0.1	0.87 ± 0.3	0.18 ± 0.3
Esterase (µmol α-naftol per µg of protein)	535.6 ± 310.8	1968.2 ± 772.8	761.03.0 ± 384.9	1635.8 ± 681.0	631.7 ± 230.2	860.5 ± 341.5
***C. cladosporioides* CBMAI 2075**	**MM1**	**MM2**	**PDB**	**MM1**	**MM2**	**PDB**
Lipase (UA)	0.97 ± 0.4	0.64 ± 0.3	0.83 ± 0.0	0.69 ± 0.2	1.47 ± 0.4	0.87 ± 0.0
Esterase (µmol α-naftol per µg of protein)	1239.78 ± 727.3	1.91 ± 1.49	1034.1 ± 437.28	392.3 ± 278.4	30.35 ± 28.83	139.1 ± 75.9

(Results expressed as mean ± standard deviation, n = 3). Fermentation time = 40 days, 28 °C at 150 rpm. PET1 = PET water bottle fragments (Crystal^®^); PET2 = PET soft drink bottle fragments (Sprite^®^); MM1 = mineral medium supplemented with glucose and sucrose; MM2 = mineral medium supplemented with palm oil; and PDB = Potato dextrose broth (Sigma^®^).

**Table 7:** Chemical modifications to PET2 after fermentation with *T. atroviride* and *C. cladosporioides* using FTIR analysis.

Wavelength in cm^−1^	*T. atroviride* CBMAI 2073	*C. cladosporioides* CBMAI 2075
3000–2500	Increased absorption intensity with the disappearance of bands between 2980 cm^−1^ and 2953 cm^−1^	No changes
2500–2000	No changes	No changes
2000–1500	Disappearance of bands at 1764 cm^−1^ and 1666 cm^−1^, intensity reduction in band at 1579 cm^−1^, and increase in absorption intensity of bands at 1502 cm^−1^ and 1500 cm^−1^	Increased absorption intensity of the bands at 1579 cm^−1^, 1573 cm^−1^, 1510 cm^−1^, and 1508 cm^−1^
1500–1000	Disappearance of bands at 1481 cm^−1^ and 1008 cm^−1^ Intensity reduction in the band at 1427 cm^−1^	No changes
1000–500	Disappearance of bands at 983 cm^−1^, 962 cm^−1^, 835 cm^−1^, and 507 cm^−1^	Intensity reduction in bands at 860 cm^−1^ and increased absorption intensity of the band at 740 cm^−1^
500–400	Disappearance of bands at 499 cm^−1^, 437 cm^−1^, and 428 cm^−1^	Disappearance of band at 437 cm^−1^

**Table 8:** Lipase and esterase activities in the fermented broth.

Enzymatic Activities in Fermented Broth Per Strain	PET1	PET2
***C. trifolii* CBMAI 2111**		
Lipase (UA)	0.31 ± 0.2	0.47 ± 0.1
Esterase (µmol α-naphthol per µg of protein)	0.55 ± 0.2	1.04 ± 0.4
***Trichoderma* sp. CBMAI 2071**		
Lipase (UA)	0.6 ± 0.1	0.42 ± 0.1
Esterase (µmol α-naftol per µg of protein)	0.48 ± 0.1	2.37 ± 0.5

Fermentation time = 15 days, 28 °C at 150 rpm; culture medium: Potato dextrose broth; results expressed as media ± standard deviation, n = 3.

**Table 9:** Polymeric modifications on PET1 and PET2 after fermentation with *T.* sp. and *C. trifolii* using FTIR analysis.

Wavelength in cm^−1^	*Trichoderma* sp. CBMAI 2071	*C. trifolii* CBMAI 2111	*Trichoderma* sp. CBMAI 2071	*C. trifolii* CBMAI 2111
PET 1			PET 2	
4000–3000	Disappearance of peaks at 3645 cm^−1^ and 3626 cm^−1^, absorption intensity increase in peaks at 3341 cm^−1^, 3334 cm^−1^, and 3226 cm^−1^	Disappearance of peaks at 3645 cm^−1^ and 3626 cm^−1^, absorption intensity increase in peaks at 3341 cm^−1^, 3334 cm^−1^, and 3226 cm^−1^	Absorption intensity increase in peak at 3646 cm^−1^	No changes
3000–2500	Absorption intensity increase in peaks at 2974 cm^−1^, 2904 cm^−1^, 2276 cm^−1^, 2389 cm^−1^, and 2340 cm^−1^, appearance of a new peak at 2540 cm^−1^	Absorption intensity increase in peaks at 2974 cm^−1^, 2904 cm^−1^, 2276 cm^−1^, 2389 cm^−1^, and 2340 cm^−1^	No changes	Absorption intensity reduction in peak at 2740 cm^−1^
2500–2000	No changes	No changes	Absorption intensity increase in peaks at 2380 cm^−1^, 2264 cm^−1^, and 2112 cm^−1^	Disappearance of peaks at 2264 cm^−1^
2000–1500	Absorption intensity increase in band at 1504 cm^−1^	Absorption intensity increase in band at 1504 cm^−1^	Absorption intensity increase in band at 1502 cm^−1^	No changes
1000–500	No changes	Appearance of a new band at 658 cm^−1^	Increase in absorption intensity increase in peaks at 956 cm^−1^ and 630 cm^−1^, disappearance of band at 656 cm^−1^	Disappearance of band at 656 cm^−1^, absorption intensity reduction in band at 956 cm^−1^

## Data Availability

The data that support the findings of this study are available on request from the corresponding author.

## Supplementary Materials

The following supporting information can be downloaded at: https://www.mdpi.com/article/10.3390/polym15061581/s1, Figure S1. The thermograms for PET1 (Crystal brand) and PET2 (Sprite brand) fragments, with melting temperatures of 251.12 °C for PET1 and 253.41 °C for PET2; Figure S2. Infrared absorption spectra for Polycaprolactone (in dichloromethane) and powdered cutin extracted from Fuji apple peels. The samples were prepared in KBr tablets; Figure S3A. FTIR spectra of the 4000 cm^−1^ to 3000 cm^−1^ region of the treatment with the isolate *Trichoderma* sp. CBMAI 2073 in PET1; Figure S3B. FTIR spectra of the 3000 cm^−1^ to 2000 cm^−1^ region of the treatment with the isolate *Trichoderma* sp. CBMAI 2073 in PET1; Figure S3C. FTIR spectra of the 2000 cm^−1^ to 1000 cm^−1^ region of the treatment with the isolate *Trichoderma* sp. CBMAI 2073 in PET1; Figure S3D. FTIR spectra of the 1000 cm^−1^ to 400 cm^−1^ region of the treatment with the isolate *Trichoderma* sp. CBMAI 2073 in PET1; Figure S3E. FTIR spectra of the 4000 cm^−1^ to 3000 cm^−1^ region of the treatment with the isolate *Trichoderma* sp. CBMAI 2073 in PET2; Figure S3F. FTIR spectra of the 3000 cm^−1^ to 2000 cm^−1^ region of the treatment with the isolate *Trichoderma* sp. CBMAI 2073 in PET2; Figure S3G. FTIR spectra of the 2000 cm^−1^ to 1000 cm^−1^ region of the treatment with the isolate *Trichoderma* sp. CBMAI 2073 in PET2; Figure S3H. FTIR spectra of the 1000 cm^−1^ to 400 cm^−1^ region of the treatment with the isolate *Trichoderma* sp. CBMAI 2073 in PET2; Figure S3I. FTIR spectra of the 4000 cm^−1^ to 3000 cm^−1^ region of the treatment with the isolate *Cladosporium cladosporioides* CBMAI 2075 in PET1; Figure S3J. FTIR spectra of the 3000 cm^−1^ to 2000 cm^−1^ region of the treatment with the isolate *Cladosporium cladosporioides* CBMAI 2075 in PET1; Figure S3K. FTIR spectra of the 2000 cm^−1^ to 1000 cm^−1^ region of the treatment with the isolate *Cladosporium cladosporioides* CBMAI 2075 in PET1; Figure S3L. FTIR spectra of the 1000 cm^−1^ to 400 cm^−1^ region of the treatment with the isolate *Cladosporium cladosporioides* CBMAI 2075 in PET1; Figure S3M. FTIR spectra of the 4000 cm^−1^ to 3000 cm^−1^ region of the treatment with the isolate *Cladosporium cladosporioides* CBMAI 2075 in PET2; Figure S3N. FTIR spectra of the 3000 cm^−1^ to 2000 cm^−1^ region of the treatment with the isolate *Cladosporium cladosporioides* CBMAI 2075 in PET2; Figure S3O. FTIR spectra of the 2000 cm^−1^ to 1000 cm^−1^ region of the treatment with the isolate *Cladosporium cladosporioides* CBMAI 2075 in PET2; Figure S3P. FTIR spectra of the 1000 cm^−1^ to 400 cm^−1^ region of the treatment with the isolate *Cladosporium cladosporioides* CBMAI 2075 in PET2; Figure S4A. FTIR spectra of the 4000 cm^−1^ to 3000 cm^−1^ region of the treatment with the isolates Curvularia trifolii CBMAI 2111 and *Trichoderma atroviride* CBMAI 2071 in PET1; Figure S4B. FTIR spectra of the 3000 cm^−1^ to 2000 cm^−1^ region of the treatment with the isolates *Curvularia trifolii* CBMAI 2111 and *Trichoderma atroviride* CBMAI 2071 in PET1; Figure S4C. FTIR spectra of the 2000 cm^−1^ to 1000 cm^−1^ region of the treatment with the isolates *Curvularia trifolii* CBMAI 2111 and *Trichoderma atroviride* CBMAI 2071 in PET1; Figure S4D. FTIR spectra of the 1000 cm^−1^ to 400 cm^−1^ region of the treatment with the isolates *Curvularia trifolii* CBMAI 2111 and *Trichoderma atroviride* CBMAI 2071 in PET1; Figure S4E. FTIR spectra of the 4000 cm^−1^ to 3000 cm^−1^ region of the treatment with the isolates *Curvularia trifolii* CBMAI 2111 and *Trichoderma atroviride* CBMAI 2071 in PET2; Figure S4F. FTIR spectra of the 3000 cm^−1^ to 2000 cm^−1^ region of the treatment with the isolates *Curvularia trifolii* CBMAI 2111 and *Trichoderma atroviride* CBMAI 2071 in PET2; Figure S4G. FTIR spectra of the 2000 cm^−1^ to 1000 cm^−1^ region of the treatment with the isolates *Curvularia trifolii* CBMAI 2111 and *Trichoderma atroviride* CBMAI 2071 in PET2; Figure S4H. FTIR spectra of the 1000 cm^−1^ to 400 cm^−1^ region of the treatment with the isolates *Curvularia trifolii* CBMAI 2111 and *Trichoderma atroviride* CBMAI 2071 in PET2; Table S1. Gradient programming applied to the chromatographic separations; Table S2. PET nanoparticle conversion (%) results after fifteen days of analysis; Table S3. Lipase and esterase activities determined by HTS with esterified fluorescent probes (results expressed as mean ± standard deviation, n = 4, analysis time of 96 h)
