# Supplementary material for: Fungal Screening for Potential PET Depolymerization
Source: Polymers (Basel). 2023 Mar 22;15(6):1581. doi: 10.3390/polym15061581 (PMC10053415; doi:10.3390/polym15061581)
Supplement: Supplementary file 1 [file polymers-15-01581-s001.zip › polymers-2128250-supplementary.pdf]

Supplementary material

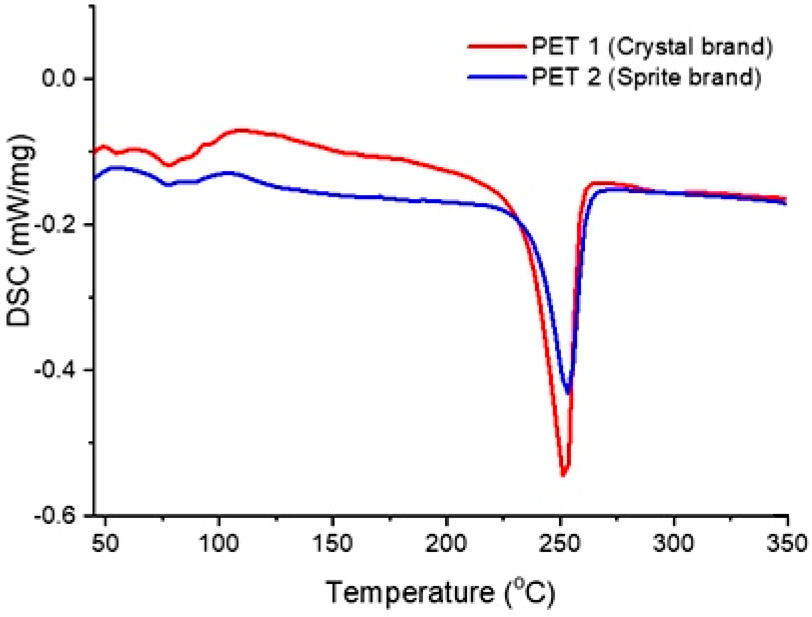

Figure S1. The thermograms for PET1 (Crystal brand) and PET2 (Sprite brand) fragments, with melting temperatures of 251.12 °C for PET1 and 253.41 °C for PET2.

$$Xc = \frac{\Delta Hm}{\Delta Hmc} \times 100Xc = \frac{\Delta Hm}{\Delta Hmc} \times 100Xc = \frac{\Delta Hm}{\Delta Hmc} \times 100 \quad \text{(S1)}$$

Equation S1. Percentage calculation of the crystallinity of the samples, where ΔHm is the sample melting enthalpy and ΔHmc is the melting heat of crystalline PET.

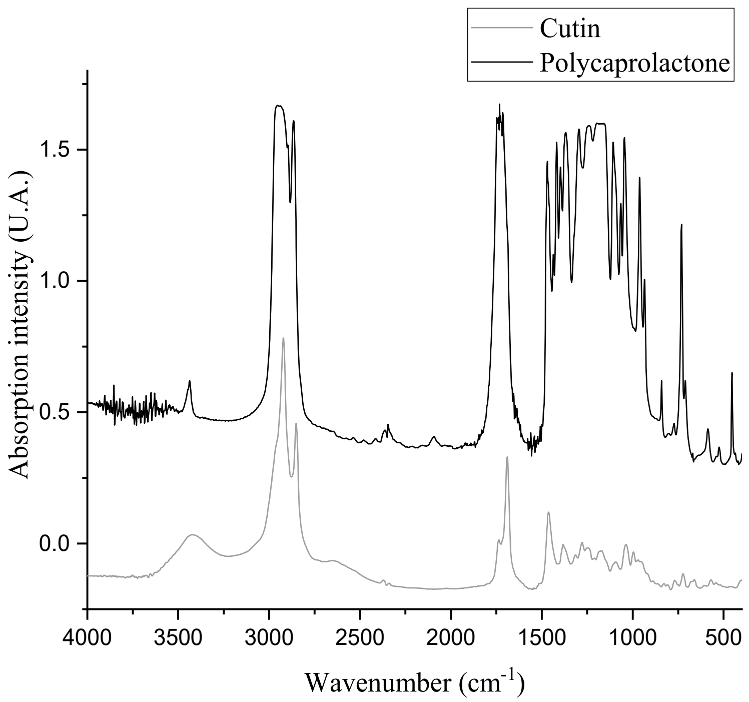

Figure S2. Infrared absorption spectra for Polycaprolactone (in dichloromethane) and powdered cutin extracted from *Fuji* apple peels. The samples were prepared in KBr tablets.

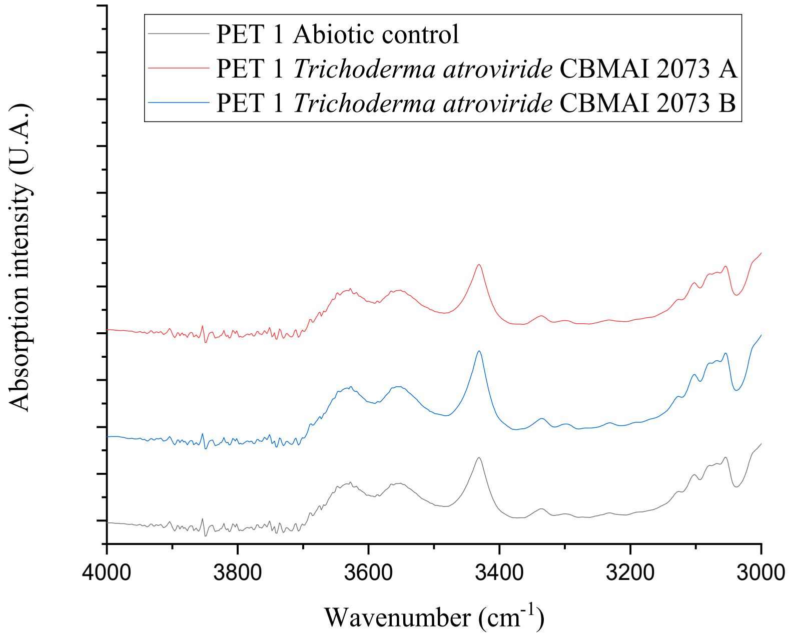

Figure S3. A. FTIR spectra of the 4000 cm^{-1} to 3000 cm^{-1} region of the treatment with the isolate *Trichoderma* sp CBMAI 2073 in PET1.

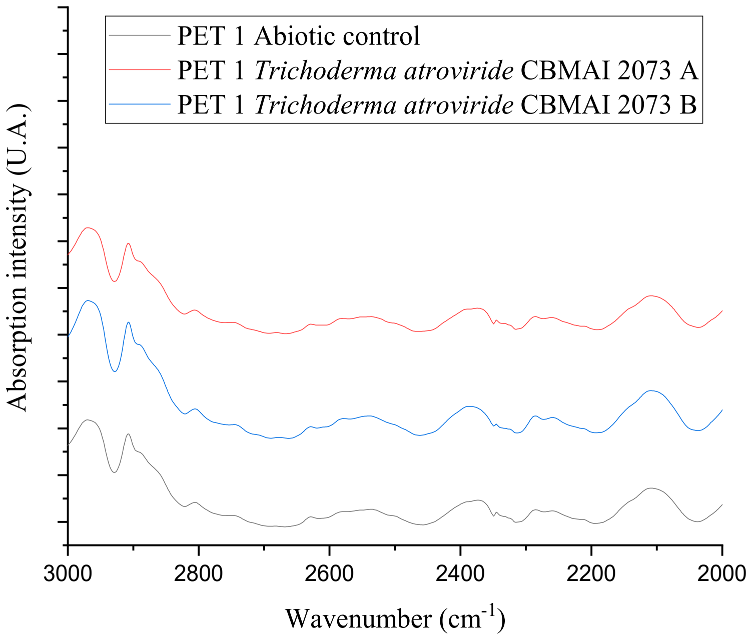

Figure S3. B. FTIR spectra of the 3000 cm^{-1} to 2000 cm^{-1} region of the treatment with the isolate *Trichoderma* sp CBMAI 2073 in PET1.

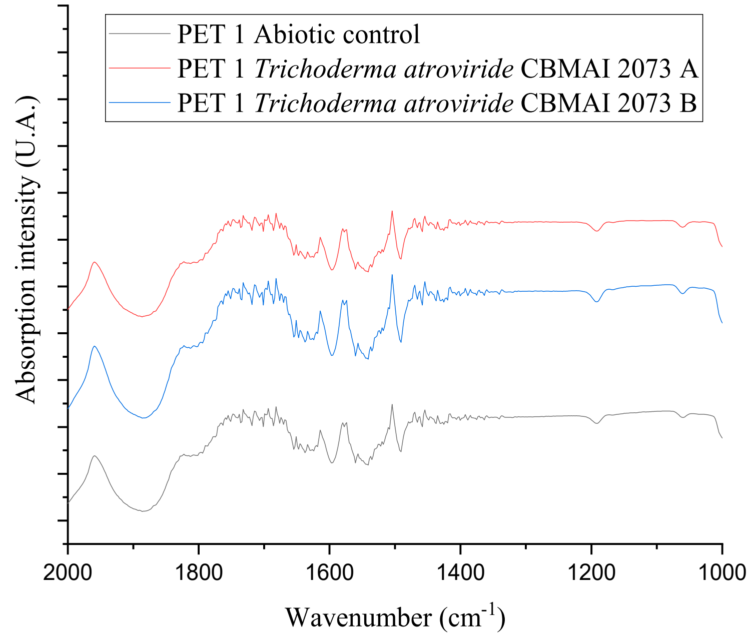

Figure S3. C. FTIR spectra of the 2000 cm^{-1} to 1000 cm^{-1} region of the treatment with the isolate *Trichoderma* sp CBMAI 2073 in PET1.

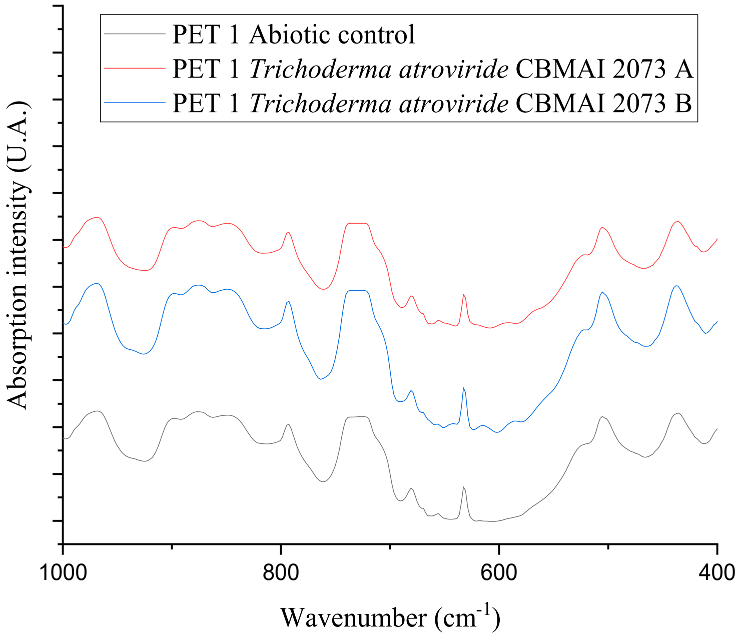

Figure S3. D. FTIR spectra of the 1000 cm^{-1} to 400 cm^{-1} region of the treatment with the isolate *Trichoderma* sp CBMAI 2073 in PET1.

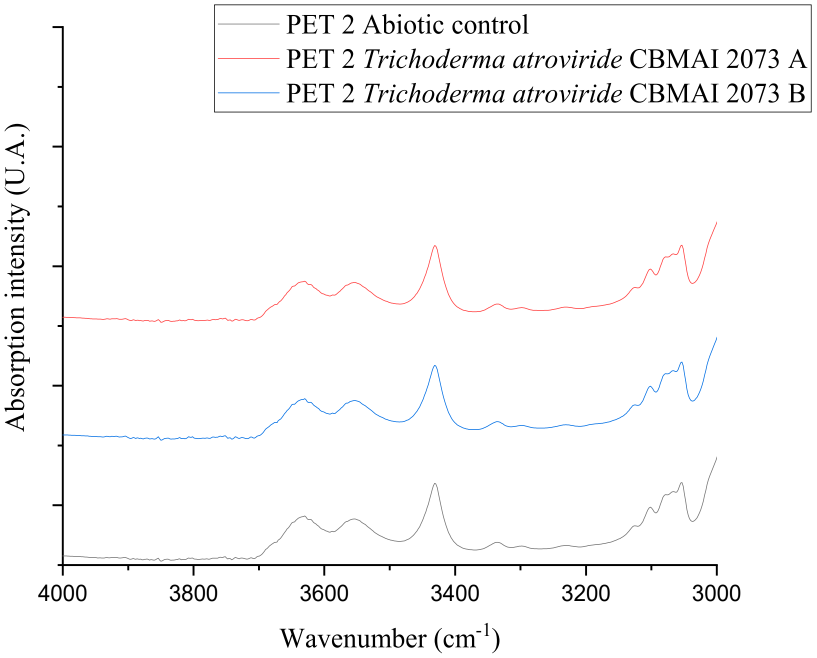

Figure S3. E. FTIR spectra of the 4000 cm^{-1} to 3000 cm^{-1} region of the treatment with the isolate *Trichoderma* sp CBMAI 2073 in PET2.

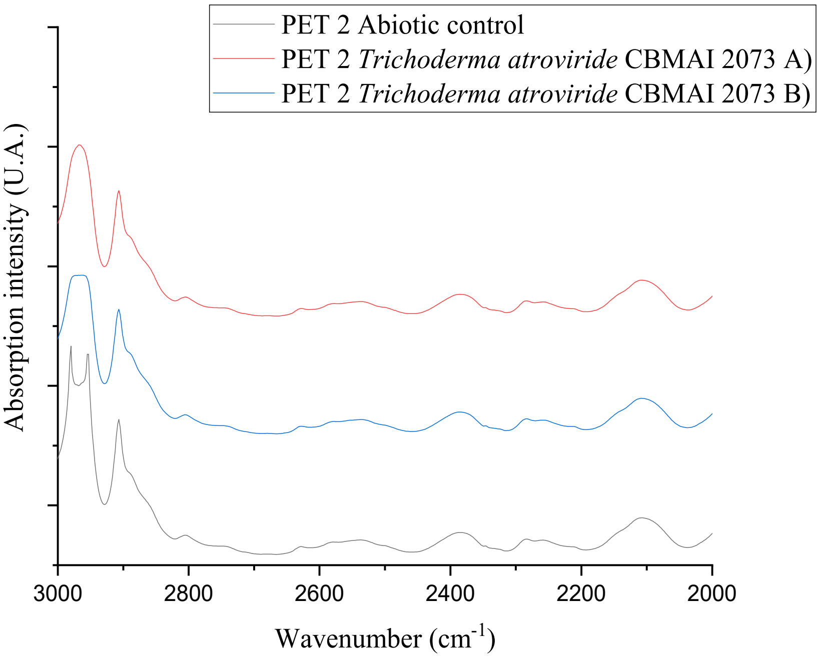

Figure S3. F. FTIR spectra of the 3000 cm^{-1} to 2000 cm^{-1} region of the treatment with the isolate *Trichoderma* sp CBMAI 2073 in PET2.

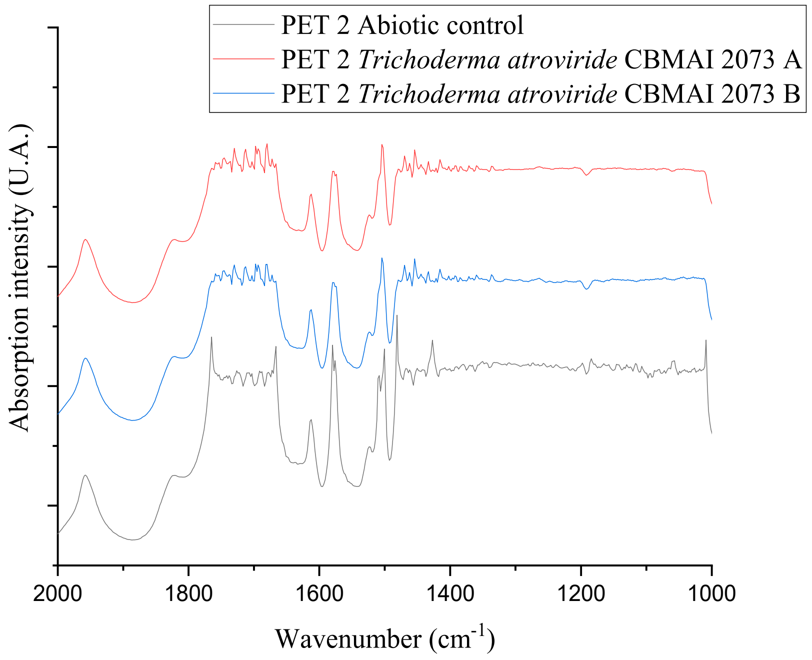

Figure S3. G. FTIR spectra of the 2000 cm^{-1} to 1000 cm^{-1} region of the treatment with the isolate *Trichoderma* sp CBMAI 2073 in PET2.

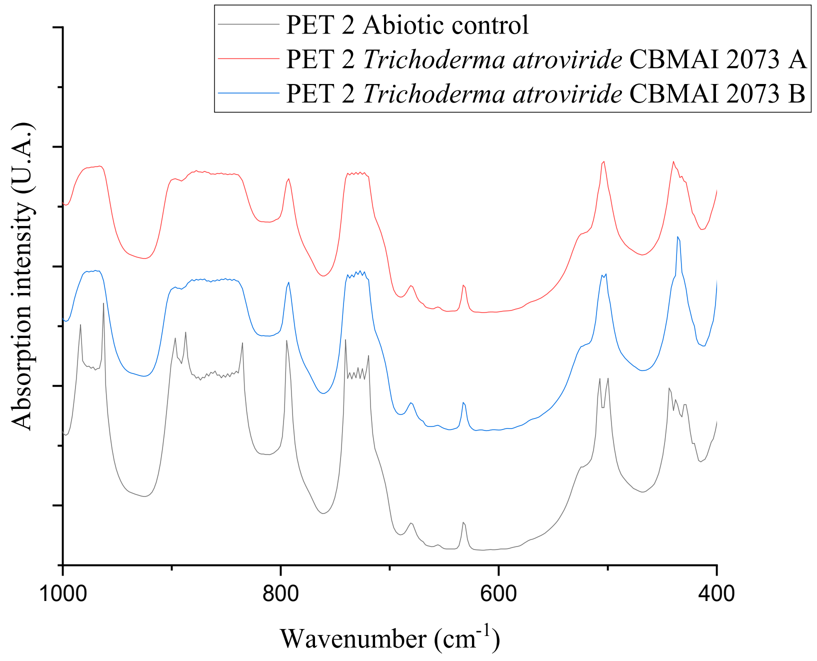

Figure S3. H. FTIR spectra of the 1000 cm^{-1} to 400 cm^{-1} region of the treatment with the isolate *Trichoderma* sp CBMAI 2073 in PET2.

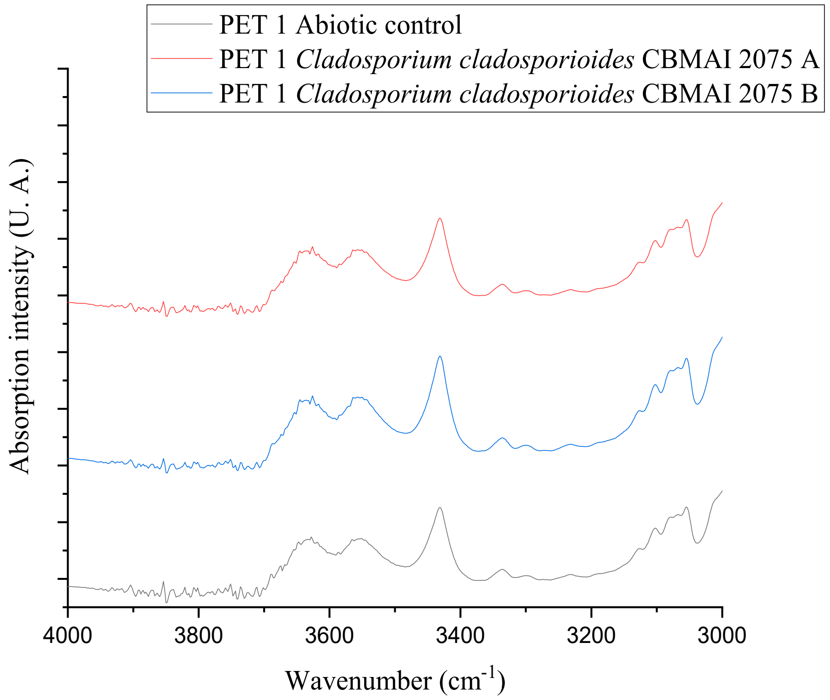

Figure S3. I. FTIR spectra of the 4000 cm^{-1} to 3000 cm^{-1} region of the treatment with the isolate *Cladosporium cladosporioides* CBMAI 2075 in PET1.

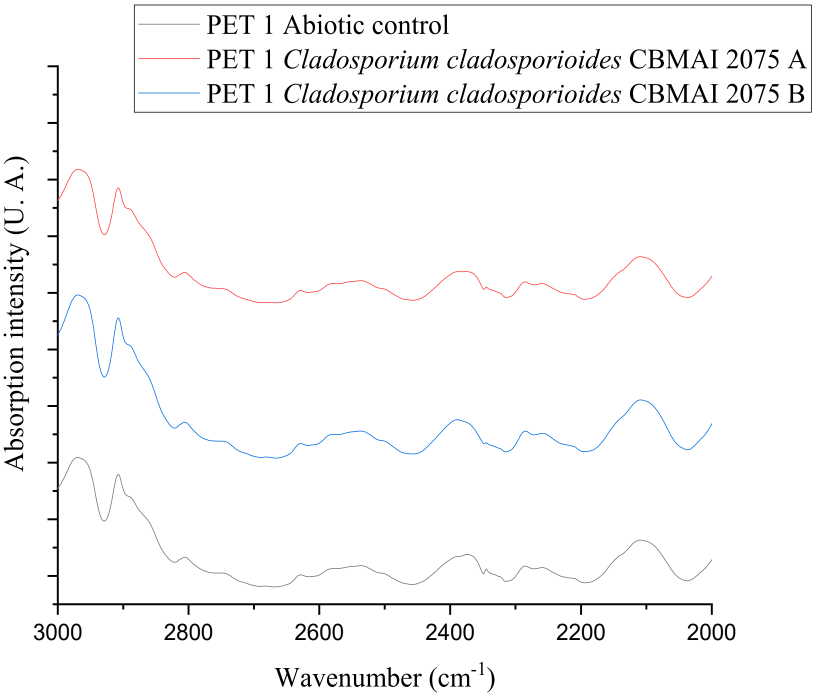

Figure S3. J. FTIR spectra of the 3000 cm^{-1} to 2000 cm^{-1} region of the treatment with the isolate *Cladosporium cladosporioides* CBMAI 2075 in PET1.

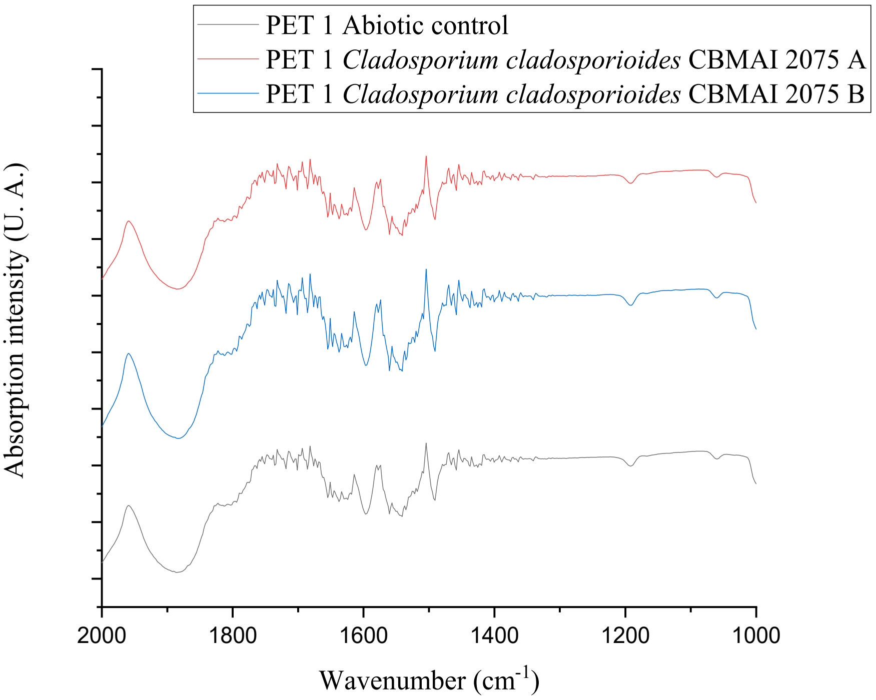

Figure S3. K. FTIR spectra of the 2000 cm^{-1} to 1000 cm^{-1} region of the treatment with the isolate *Cladosporium cladosporioides* CBMAI 2075 in PET1.

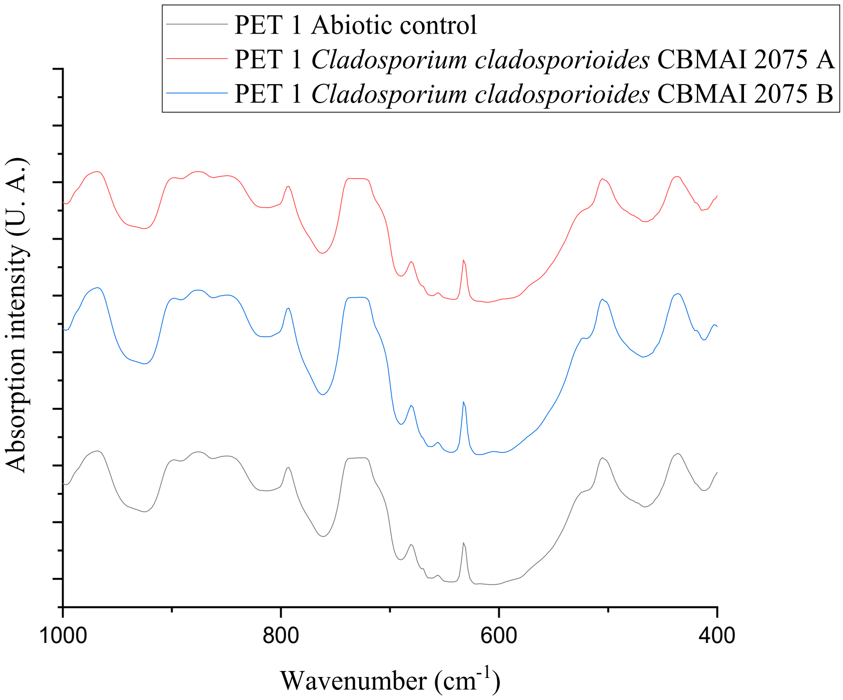

Figure S3. L. FTIR spectra of the 1000 cm^{-1} to 400 cm^{-1} region of the treatment with the isolate *Cladosporium cladosporioides* CBMAI 2075 in PET1.

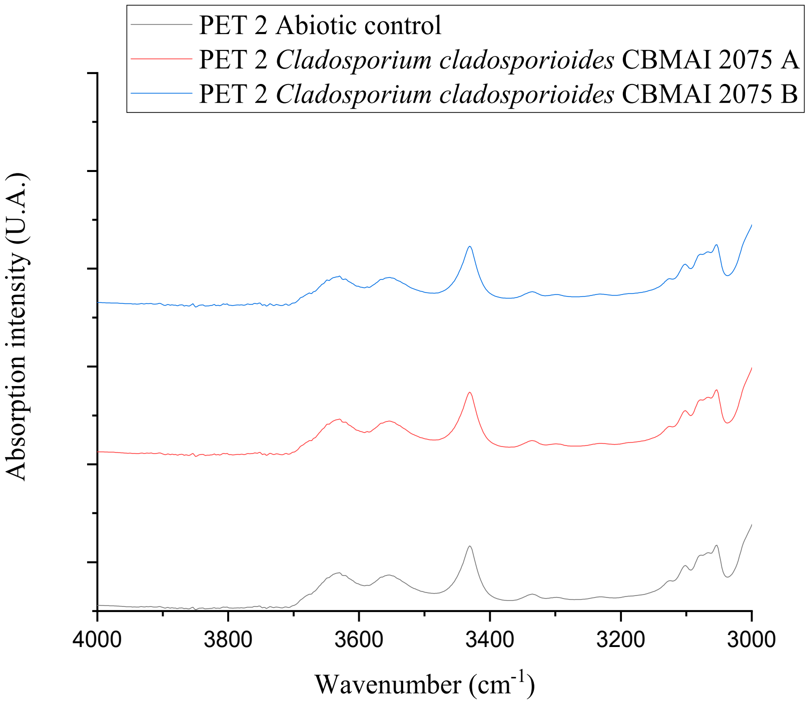

Figure S3. M. FTIR spectra of the 4000 cm^{-1} to 3000 cm^{-1} region of the treatment with the isolate *Cladosporium cladosporioides* CBMAI 2075 in PET2.

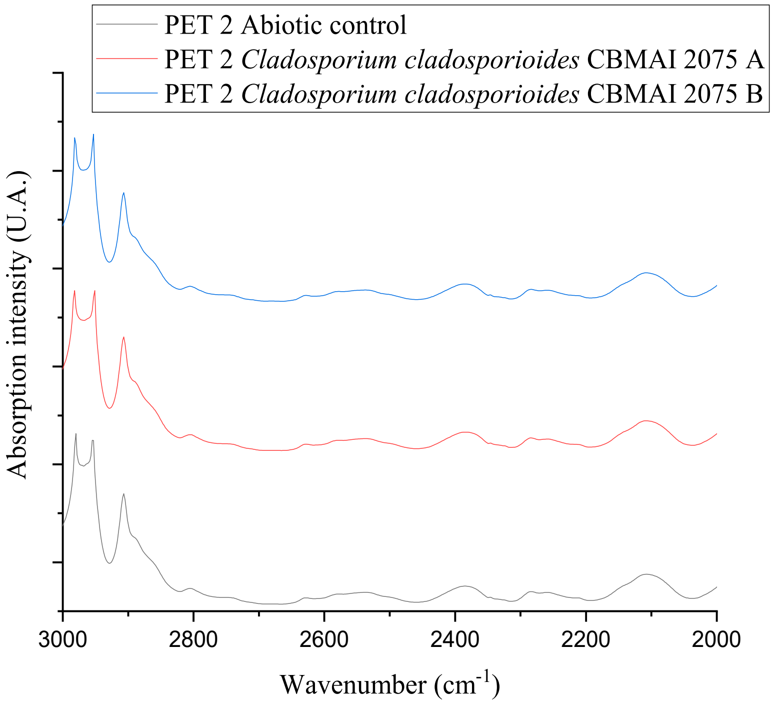

Figure S3. N. FTIR spectra of the 3000 cm-1 to 2000 cm-1 region of the treatment with the isolate *Cladosporium cladosporioides* CBMAI 2075 in PET2.

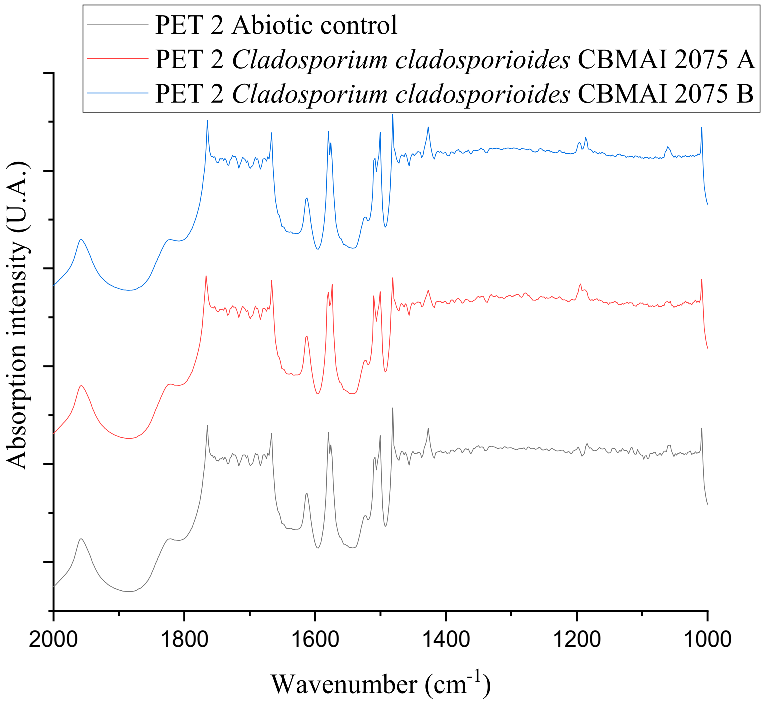

Figure S3. O. FTIR spectra of the 2000 cm-1 to 1000 cm-1 region of the treatment with the isolate *Cladosporium cladosporioides* CBMAI 2075 in PET2.

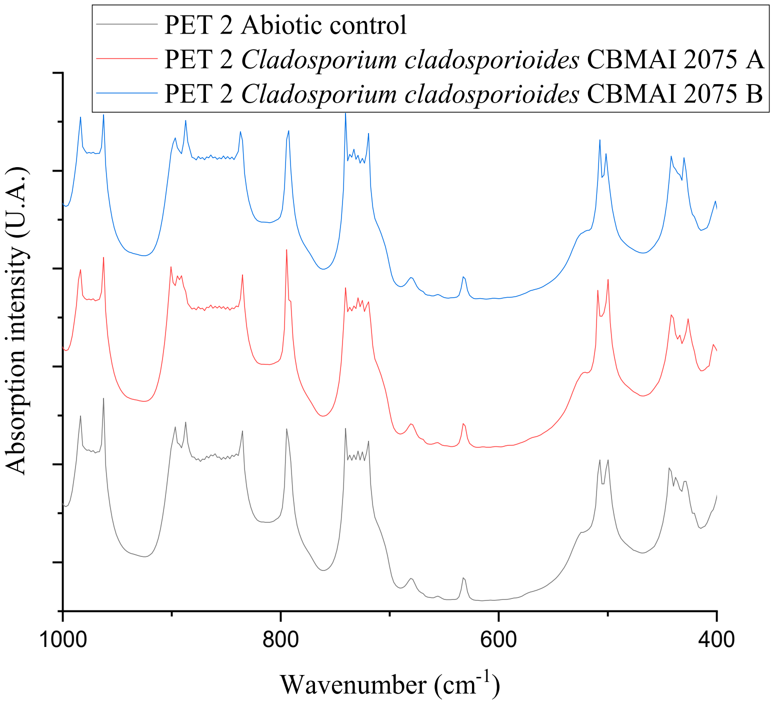

Figure S3. P. FTIR spectra of the 1000 cm^{-1} to 400 cm^{-1} region of the treatment with the isolate *Cladosporium cladosporioides* CBMAI 2075 in PET2.

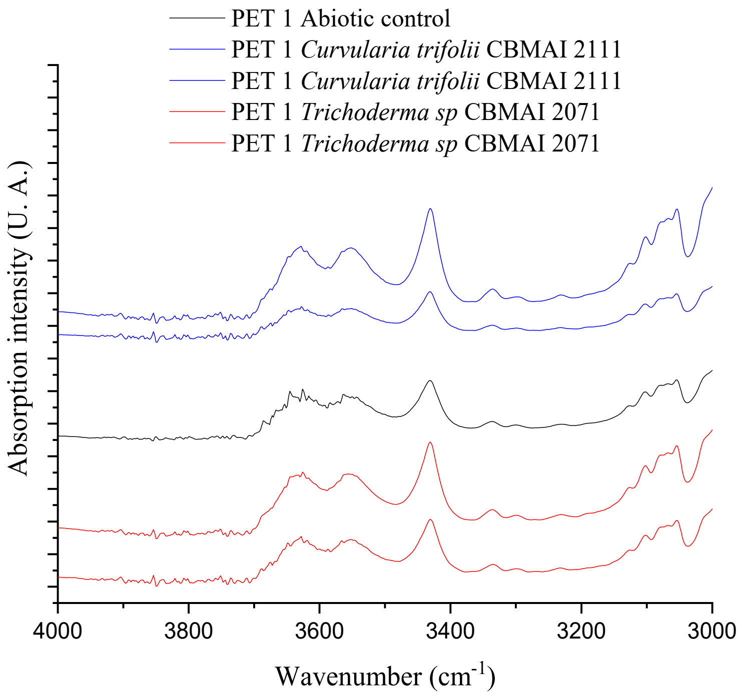

Figure S4. A. FTIR spectra of the 4000 cm^{-1} to 3000 cm^{-1} region of the treatment with the isolates *Curvularia trifolii* CBMAI 2111 and *Trichoderma atroviride* CBMAI 2071 in PET1.

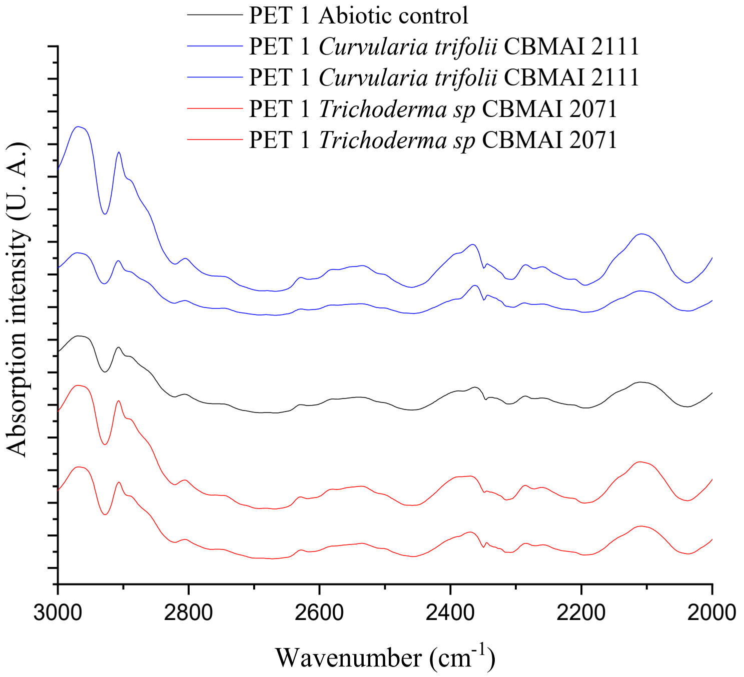

Figure S4. B. FTIR spectra of the 3000 cm^{-1} to 2000 cm^{-1} region of the treatment with the isolates *Curvularia trifolii* CBMAI 2111 and *Trichoderma atroviride* CBMAI 2071 in PET1.

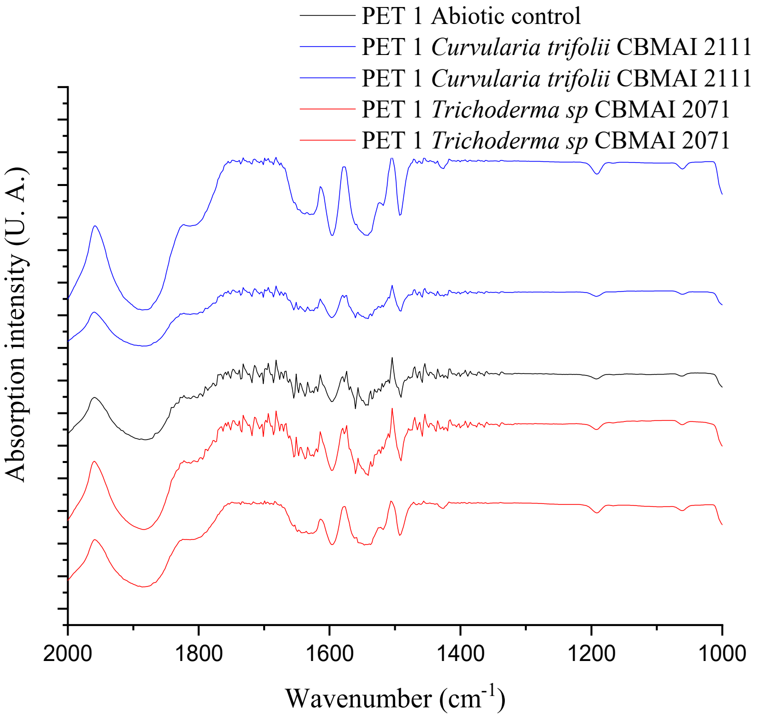

Figure S4. C. FTIR spectra of the 2000 cm^{-1} to 1000 cm^{-1} region of the treatment with the isolates *Curvularia trifolii* CBMAI 2111 and *Trichoderma atroviride* CBMAI 2071 in PET1.

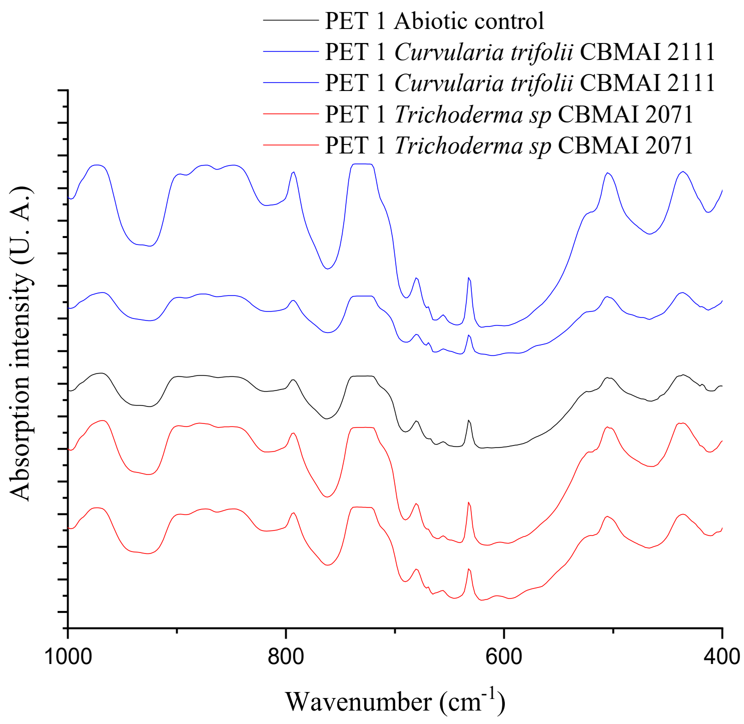

Figure S4. D. FTIR spectra of the 1000 cm^{-1} to 400 cm^{-1} region of the treatment with the isolates *Curvularia trifolii* CBMAI 2111 and *Trichoderma atroviride* CBMAI 2071 in PET1.

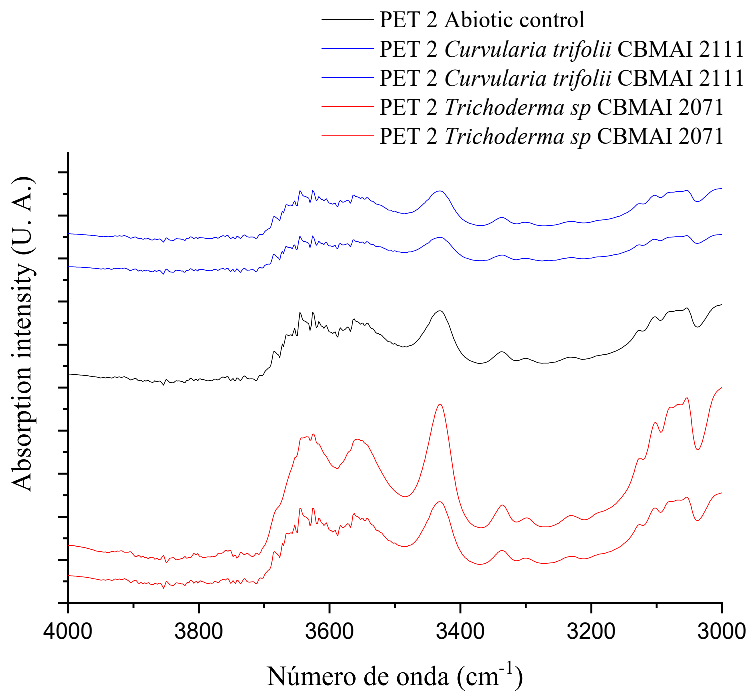

Figure S4. E. FTIR spectra of the 4000 cm^{-1} to 3000 cm^{-1} region of the treatment with the isolates *Curvularia trifolii* CBMAI 2111 and *Trichoderma atroviride* CBMAI 2071 in PET2.

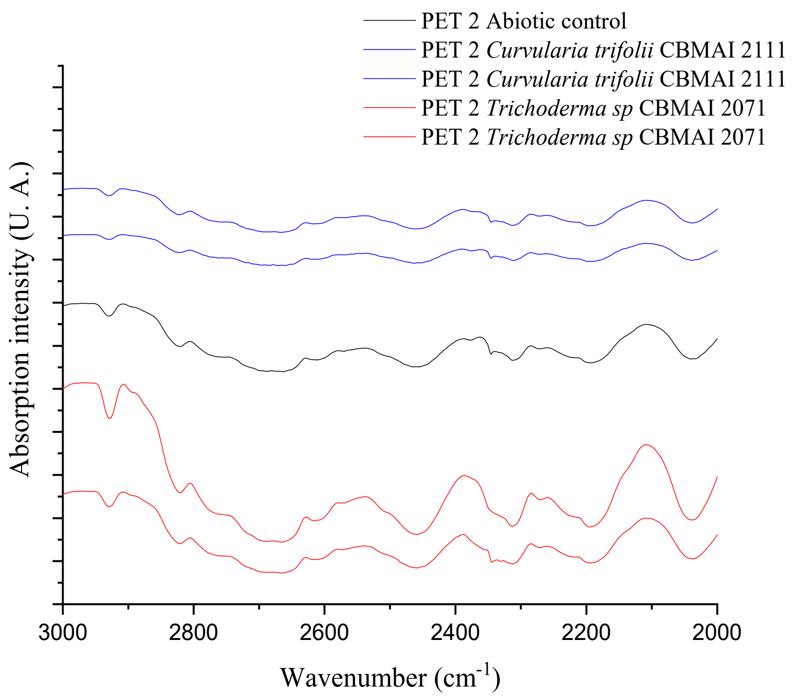

Figure S4. F. FTIR spectra of the 3000 cm^{-1} to 2000 cm^{-1} region of the treatment with the isolates *Curvularia trifolii* CBMAI 2111 and *Trichoderma atroviride* CBMAI 2071 in PET2.

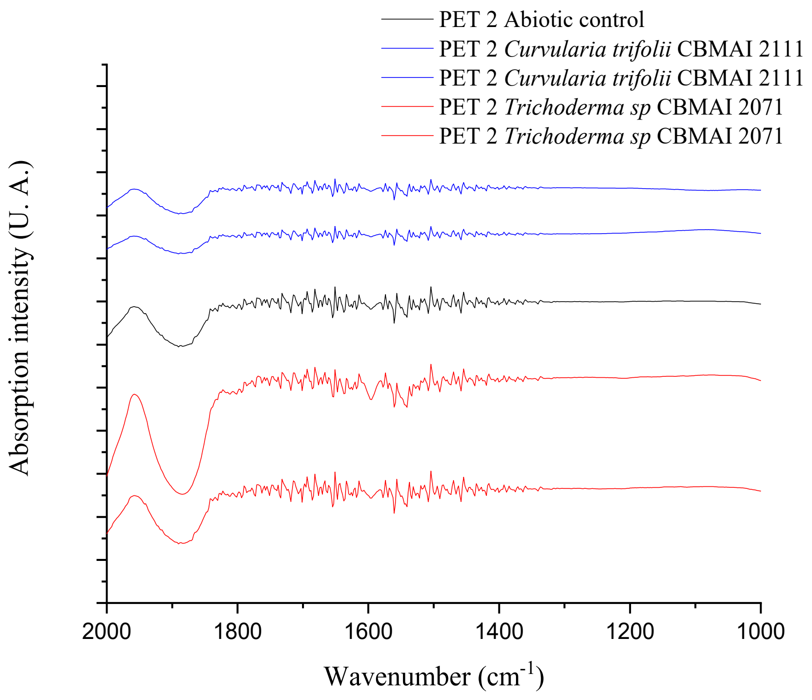

Figure S4. G. FTIR spectra of the 2000 cm^{-1} to 1000 cm^{-1} region of the treatment with the isolates *Curvularia trifolii* CBMAI 2111 and *Trichoderma atroviride* CBMAI 2071 in PET2.

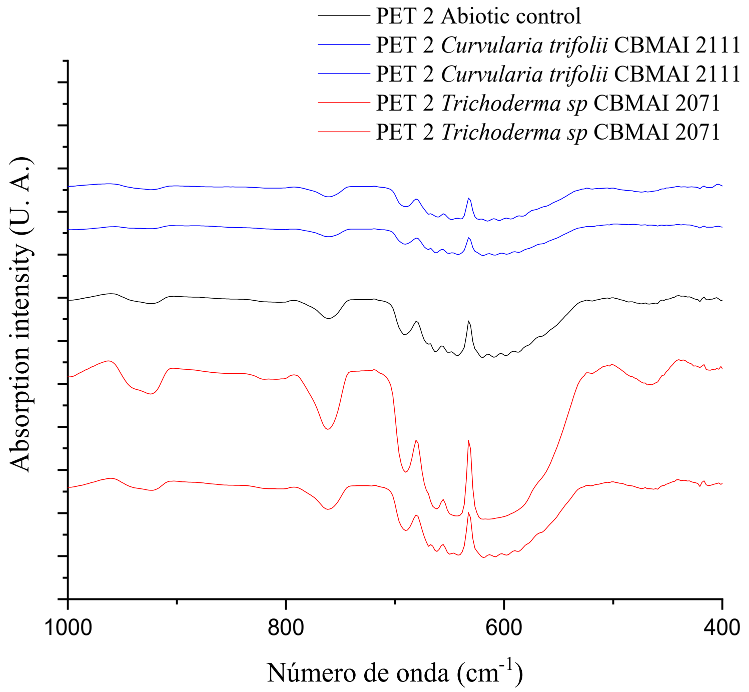

Figure S4. H. FTIR spectra of the 1000 cm^{-1} to 400 cm^{-1} region of the treatment with the isolates *Curvularia trifolii* CBMAI 2111 and *Trichoderma atroviride* CBMAI 2071 in PET2.

Table S1. Gradient programming applied to the chromatographic separations.

Time (minutes)	Mobile phase B (%)
**0.01**	80
**5.0**	40
**13.0**	50
**16.0**	0
**24.0**	0
**25.0**	80
**30.0**	80
**35.0**	80
**40.0**	80

Table S2. PET nanoparticle conversion (%) results after fifteen days of analysis.

Code	Identification	NPPET conversion (%)*
**CBMAI 2111**	***Curvularia trifolii***	**9.0±1.1**
**CBMAI 2073**	***Trichoderma atroviride***	**6.1±0.2**
**CBMAI 2071**	***Trichoderma sp.***	**3.6±0.8**
CBMAI 2111	*Microsphaeropsis arundinis*	2.7±0.9
CBMAI 2109	*Microsphaeropsis arundinis*	2.0±0.4
LMA 1269	*Fusarium* sp.	1.9±0.5
CBMAI 2159	*Pseudallescheria* sp. (Pseudallescheria/Scedosporium complex)	1.9±1.0
CBMAI 2083	*Paecilomyces* sp.	1.7±0.7
CBMAI 2189	*Aspergillus fumigatus*	0.8±0.6
**CBMAI 2075**	***Cladosporium cladosporioides***	**0.8±0.6**
LMA1251	*Trichoderma* sp.	0.7±0.4
CBMAI 2158	*Paraconiothyrium cyclothyrioides*	0.7±0.1
CBMAI 2191	*Penicillium koreense*	0.7±0.1
CBMAI 2203	*Paraconiothyrium cyclothyrioides*	0.6±0.3
LMA 216	*Phoma herbarum*	0.6±0.6
LMA 28	*Aspergillus fumigatus*	0.5±0.0
LMA 1825	*Fusarium* sp.	0.4±0.0

LMA 1172	*Fusarium* sp.	0.4±0.0
CBMAI 2190	*Aspergillus fumigatus*	0.4±0.2
CBMAI 2186	*Talaromyces veerkampii*	0.3±0.2
CBMAI 2187	*Paraconiothyrium cyclothyrioides*	0.3±0.3
CBMAI 2149	*Trichoderma capillare*	0.3±0.0
CBMAI 2155	*Microsphaeropsis arundinis*	0.3±0.2
LMA 167	*Penicillium* sp.	0.3±0.1
LMA 11	*Paecilomyces-like*	0.2±0.0
LMA 1145	*Fusarium* sp.	0.1±0.0

*conversion results from NPPET into HOTP expressed as mean percentage ± standard deviation (n = 3)

Table S3. Lipase and esterase activities determined by HTS with esterified fluorescent probes (results expressed as mean ± standard deviation, n = 4, analysis time of 96 h).

**Strain**	**Conversion % (2-carbon probe)**	**Conversion % (8-carbon probe)**
C. trifolii CBMAI 2111	11.9±0.1	10.8±2.1
T. atroviride CBMAI 2073	37.2±4.9	46.0±3.4
Trichoderma sp. CBMAI 2071	0.0±0.0	0.0±0.0
C. cladosporioides CBMAI 2075	41.66±0.8	41.2±0.9
